# Supplementary material for: Development of an occupational advice intervention for patients undergoing elective hip and knee replacement: a Delphi study
Source: BMJ Open. 2020 Jul 6;10(7):e036191. doi: 10.1136/bmjopen-2019-036191 (PMC7342851; doi:10.1136/bmjopen-2019-036191)
Supplement: Supplementary data [file bmjopen-2019-036191supp001.pdf]

Appendices

	Category	Statements reaching consensus
1	Responsibility for delivery and co-ordination of the return to work intervention	Individual orthopaedic surgeons should be responsible for co-ordinating the return to work intervention for their own patients (88% disagreement)
2	Pre-operative identification of patients at 'higher risk' of prolonged sickness absence following surgery that may require additional individualised help and support	A face-to-face assessment with a staff member trained in the return to work intervention should be used to identify patients at 'higher risk' of prolonged sickness absence following surgery (80% agreement)
3	Pre-operative needs assessment	Hospital physiotherapy teams should see all patients intending to return to work prior to their surgery (80% agreement)<br><br>Hospital occupational therapy teams should see all 'higher risk' patients intending to return to work prior to their surgery (88% agreement)<br><br>Hospital physiotherapy team should see all 'higher risk' patients intending to return to work prior to their surgery (80% agreement)<br><br>Development of a return to work plan should be a routine part of the pre-assessment process for all patients undergoing hip and knee replacement (80% agreement)<br><br>Development of a return to work plan should be a routine part of the pre-assessment process for all 'higher risk' patients undergoing hip and knee replacements (96% agreement)<br><br>Pre-operative education sessions conducted by the hospital orthopaedic team should routinely include information about returning to work after surgery (88% agreement)<br><br>Patients should be encouraged to have a meeting with their employer prior to surgery to discuss their recovery and plans for returning to work (100% agreement)
4	Post-operative identification of patients at risk of an extended period off work after surgery	All return to work patients should be offered a routine physiotherapy review after discharge from hospital to help monitor their progress and identify those patients at risk of an extended period off work after surgery (84% agreement)<br><br>All return to work patients should be contacted by a member of the therapy team after discharge to help monitor their progress and identify those patients at risk of an extended period off work after surgery (92% agreement)<br><br>The hospital should provide a means of contact (phone, email etc) through which patients in need of additional help and support can contact the hospital orthopaedic team (92% agreement)<br><br>Patients should be encouraged to have a meeting with their employer in the 4 weeks following surgery to discuss their recovery and plan for return to work (72% agreement)
5	Additional care for Patients identified as 'higher risk' of an extended period off work after surgery	Additional post-operative physiotherapy review with a physiotherapist until specific work related rehabilitation targets have been achieved (92% agreement)<br><br>Additional post-operative occupational therapy review (76% agreement)<br><br>Ongoing therapy involvement until 'return to work' has been achieved

		(76% agreement)<br><br>Access to additional procedure specific information and advice (92% agreement)<br><br>Referral to the Fit4Work service (84% agreement)
6	Scope of training for staff	All members of the hospital orthopaedic team involved in the treatment of hip and knee replacement patients should receive training regarding the return to work intervention (76% agreement)<br><br>Specific staff members with responsibility for delivering 'return to work' advice should receive training regarding the return to work intervention (96% agreement)<br><br>All members of the therapy teams treating hip and knee replacement patients should receive training regarding the return to work intervention (88% agreement)<br><br>Training regarding the return to work intervention should be offered to local G.P groups (72% agreement)
7	Communicating occupational status and progress between stakeholders	The details of the patient's occupational status should be recorded in the primary care referral letter (96% agreement)<br><br>Details of the patient's occupational status should be confirmed and recorded in all interactions with secondary care (e.g. pre-operative outpatient appointments, discharge summaries post-operative follow-up appointment letters) (84% agreement)<br><br>Any information developed with the healthcare team as part of a patient return to work plan should be made available to the G.P (96% agreement)<br><br>Any information developed with the healthcare team as part of a patient return to work plan should be made available to the patient's employer and/or occupational health provider with their consent (84% agreement)<br><br>It is the patients and not the healthcare teams responsibility to communicate with their employer about their return to work (76% agreement)<br><br>All post-operative assessments by members of the therapy team should generate a statement about the patient's current functional status and estimated timeframe to return to work that is given to the patient and forwarded to their G.P (84% agreement)
8	Fit Notes	Fit notes should be completed in accordance with Department for Work and Pensions Fit Note Guidance (96% agreement)<br><br>Hospital orthopaedic departments should actively ask each and every patient if they require a fit note prior to discharge (96% agreement)<br><br>To discourage extended periods off work patients should be issued with a basic fit note excusing them from work for 2 weeks. Further fit notes can be issued based on progress towards recovery and access to phased return or amended duties and hours (72% disagreement)<br><br>G.Ps should be responsible for administering subsequent fit notes based on progress reports from the hospital orthopaedic team (76% agreement)
9	Format and delivery of patient information	All of the information and advice components relevant to patients should be included within a written patient booklet that is given to all patients. Information for other stakeholders will be delivered separately (76% agreement)<br><br>Only the most important (based on the ranking listed previously) information and advice components relevant to patients should be

		included within a written patient booklet that is given to all patients. Additional information could be made available via a website (80% agreement)<br><br>The patient booklet should include a section within which the patient and other stakeholders can record progress and feedback. This information would be held by the patient and could be shared with their permission (80% agreement)<br><br>The patient booklet should include a section within which the patient can record the outcome of any meetings and with their employer and/or occupational health team. This information would be held by the patient and could be shared with their permission (92% agreement)<br><br>Patients with previous experience of returning to work after joint replacement should be involved in the drafting of any patient facing materials (92% agreement)<br><br>The occupational advice intervention should include the ability for local hospital department to upload their own policies and procedures relating to post-operative recovery and return to work (92% agreement)<br><br>Primary Care Health professional and support staff should be able to direct potential surgical candidates to return to work information and advice prior to referral (88% agreement)
10	When should the intervention commence?	The intervention should commence in secondary care after the patient has had their operation but prior to discharge (88% disagreement)
11	Defining return to work	A patient has 'returned to work' only when they return to the same job (usual hours and duties) and activities outside of work they were doing prior to surgery (72% disagreement)
12	The aim of the intervention	The return to work interventions primary aim should be to return the patient to their pre-operative work role / level of occupational performance. (76% agreement)
13	Measuring return to work	The measurement of return to work should be graded based on specific work milestones for example return to place of work, return to normal hours, return to normal workplace activities (92% agreement)<br><br>The measurement of return to work should be based on resumption of specific work related activities (e.g. climbing, lifting, manual tasks) based on a list of pre-operative workplace activities (72% agreement)<br><br>The measurement of return to work is a binary measure – the patient has either returned to work or they have not (72% disagreement).<br><br>The rates of further sick leave in the 6 months after the patient has returned to work once is an important outcome measure (80% agreement)<br><br>The rates (type and duration) of any occupational adjustments implemented by the employer is an important outcome measure (84% agreement)<br><br>Asking patients about how they feel about their return to work is important outcome measure (96% agreement)<br><br>An assessment of whether or not the patient's preoperative occupational expectations following surgery have been met is an important outcome measure (92% agreement)

Appendix Table 1: Statements descriptions reaching consensus for Round 2
