# Supplementary material for: Development of an occupational advice intervention for patients undergoing elective hip and knee replacement: a Delphi study
Source: BMJ Open. 2020 Jul 6;10(7):e036191. doi: 10.1136/bmjopen-2019-036191 (PMC7342851; doi:10.1136/bmjopen-2019-036191)
Supplement: Author's manuscript [file bmjopen-2019-036191.draft_revisions.pdf]

BMJ Open is committed to open peer review. As part of this commitment we make the peer review history of every article we publish publicly available.

When an article is published we post the peer reviewers' comments and the authors' responses online. We also post the versions of the paper that were used during peer review. These are the versions that the peer review comments apply to.

The versions of the paper that follow are the versions that were submitted during the peer review process. They are not the versions of record or the final published versions. They should not be cited or distributed as the published version of this manuscript.

BMJ Open is an open access journal and the full, final, typeset and author-corrected version of record of the manuscript is available on our site with no access controls, subscription charges or pay-per-view fees (http://bmjopen.bmj.com).

If you have any questions on BMJ Open's open peer review process please email info.bmjopen@bmj.com

BMJ Open

Development of an occupational advice intervention for patients undergoing elective hip and knee replacement: A Delphi study

Journal:	*BMJ Open*
Manuscript ID	bmjopen-2019-036191
Article Type:	Original research
Date Submitted by the Author:	07-Dec-2019
Complete List of Authors:	Baker, Paul; South Tees Hospitals NHS Foundation Trust, Department of Orthopaedics ; University of York,<br>Kottam, Lucksy; South Tees Hospitals NHS Foundation Trust, Department of Orthopaedics<br>Coole, Carol; University of Nottingham, 3. School of Health Sciences, Faculty of Medicine & Health Sciences<br>Drummond, Avril; University of Nottingham, Div of Rehabilitation and Ageing<br>McDaid, Catriona; University of York, York Trials Unit<br>Rangan, Amar; The James Cook University Hospital, Trauma and Orthopaedics; University of York, Department of Health Sciences
Keywords:	OCCUPATIONAL & INDUSTRIAL MEDICINE, Adult orthopaedics < ORTHOPAEDIC & TRAUMA SURGERY, Hip < ORTHOPAEDIC & TRAUMA SURGERY, Knee < ORTHOPAEDIC & TRAUMA SURGERY, Musculoskeletal disorders < ORTHOPAEDIC & TRAUMA SURGERY

SCHOLARONE™
Manuscripts

I, the Submitting Author has the right to grant and does grant on behalf of all authors of the Work (as defined in the below author licence), an exclusive licence and/or a non-exclusive licence for contributions from authors who are: i) UK Crown employees; ii) where BMJ has agreed a CC-BY licence shall apply, and/or iii) in accordance with the terms applicable for US Federal Government officers or employees acting as part of their official duties; on a worldwide, perpetual, irrevocable, royalty-free basis to BMJ Publishing Group Ltd ("BMJ") its licensees and where the relevant Journal is co-owned by BMJ to the co-owners of the Journal, to publish the Work in this journal and any other BMJ products and to exploit all rights, as set out in our licence.

The Submitting Author accepts and understands that any supply made under these terms is made by BMJ to the Submitting Author unless you are acting as an employee on behalf of your employer or a postgraduate student of an affiliated institution which is paying any applicable article publishing charge ("APC") for Open Access articles. Where the Submitting Author wishes to make the Work available on an Open Access basis (and intends to pay the relevant APC), the terms of reuse of such Open Access shall be governed by a Creative Commons licence – details of these licences and which Creative Commons licence will apply to this Work are set out in our licence referred to above.

Other than as permitted in any relevant BMJ Author's Self Archiving Policies, I confirm this Work has not been accepted for publication elsewhere, is not being considered for publication elsewhere and does not duplicate material already published. I confirm all authors consent to publication of this Work and authorise the granting of this licence.

For peer review only

Title

Development of an occupational advice intervention for patients undergoing elective hip and knee replacement: A Delphi study

Authors

- Paul Baker (1,2)
- Lucksy Kottam (1)
- Carol Coole (3)
- Avril Drummond (3)
- Catriona McDaid (2)
- Amar Rangan (1,2,4)

On behalf of the OPAL study group (see acknowledgments)

Institutions

1. South Tees Hospitals NHS Foundation Trust, Middlesbrough
2. York Trials Unit, Department of Health Sciences, University of York
3. School of Health Sciences, Faculty of Medicine & Health Sciences, University of Nottingham
4. University of Oxford, Oxford

Corresponding author information

Email: paul.baker1@nhs.net
Tel: 01642 854479

For peer review only

Abstract

Objective: To obtain consensus to support the development of an occupational advice intervention for patients undergoing primary hip and knee replacement surgery.

Design: Modified Delphi study as part of a wider intervention development study (The OPAL study: HTA Reference 15/28/02).

Setting: Five stakeholder groups (patients, employers, orthopaedic surgeons, General Practitioners (GPs), allied health professionals (AHPs) and nurses) recruited from across the United Kingdom.

Participants: Sixty-six participants.

Methods: Statements for the Delphi process were developed relating to the content, format, delivery, timing and measurement of an occupational advice intervention. The statements were based on evidence gathered through the OPAL study that was processed using an intervention mapping framework. Intervention content was examined in Round 1 and intervention format, delivery, timing and measurement were examined in Round 2. In Round 3 the developed intervention was presented to the stakeholder groups for comment.

Consensus: For Rounds 1 and 2 consensus was defined as 70% agreement or disagreement on a 4-point scale. Statements reaching consensus were ranked according to the distribution of responses to create a hierarchy of agreement. Round 3 comments were used to revise the final version of the developed occupational advice intervention.

Results: Consensus was reached for 36 of 64 Round 1 content statements (all agreement). In Round 2, 13 questions were carried forward and an additional 81 statements were presented. Of these, 49 reached consensus (44 agreement/5 disagreement). Round 3 responses to the developed intervention were positive and finalised the design.

Conclusions: The Delphi process informed the development of an occupational advice intervention as part of a wider intervention development study. Stakeholder agreement was achieved for a large number of intervention elements encompassing the content, format, delivery and timing of the intervention. The effectiveness and cost-effectiveness of the developed intervention will require evaluation in a randomized controlled trial.

Keywords: Return to work, Hip replacement, Knee replacement, Occupational advice, Employment, Surgery

Strengths and limitations of this study:

- Wide stakeholder engagement including patients, employers, surgeons, GPs, Allied Health professionals with knowledge of the process of returning to work after hip or knee replacement
- Recruitment of a large number of Delphi members who were invited to participate in all 3 Delphi rounds
- Initial Delphi statements developed from research performed as part of the OPAL (Occupational advice for Patients undergoing Arthroplasty of the Lower limb) study (HTA 15/28/02).
- Loss of participants between each of the rounds of questioning, specifically the employer group who failed to participate in the final Delphi round.

For peer review only

Introduction

Hip and knee osteoarthritis are associated with a reduction in work participation and productivity and an increased risk of work loss (1,2). The costs associated with occupational musculoskeletal disorders are significant (3,4). The estimated annual cost of workplace ill health is £9.7 billion, equivalent to £18,400 per case (5). These costs are borne not only by the individual (impact of ill health on quality of life), but also by their employers and society (loss of productivity, need for health care, rehabilitation and compensation) (3,4). In addition to the financial costs, lengthy sickness absence can result in work disability, poorer general health, increased risk of mental health problems and higher mortality (6,7,8). Working therefore has physical and mental health benefits, alongside its socioeconomic value.

Lower limb joint replacement is an effective and cost-effective treatment for patients with hip and knee osteoarthritis (9,10,11,12). Recent changes to the pension age combined with an ageing UK workforce have resulted in a steady increase in the numbers of hip and knee replacements being performed in patients of working age over the last decade (13,14,15). In 2017, 18,812 (20.5% of all hip replacements) and 17,765 (17.4% of all knee replacements) were performed in patients aged less than 60 years (13,14,15). Current recommendations supporting return to work after hip and knee replacement are limited and inconsistent (16). There is variation in the content, delivery and format of occupational advice delivered to patients having hip and knee replacements and a need to provided more comprehensive, individualised advice for these patients to support early, sustained return to work after surgery (16).

The OPAL study (Occupational advice for Patients undergoing Arthroplasty of the Lower limb) was a National Institute for Health Research – Health Technology Assessment (NIHR-HTA) commissioned research study that aimed to develop an occupational advice intervention to support return to work after hip and knee replacement (16). OPAL used an intervention mapping framework supported by related qualitative and quantitative work streams (16). Initial research evaluated the specific needs of the population of patients who were in work and intended to return to work following surgery, established how individual patients returned to work and documented the barriers preventing return to work (17,18). Through these work streams a range of key performance indicators and potential intervention components that could be used to develop an occupational advice intervention emerged.

To refine these components and address areas of uncertainty relating to the intervention a multi-stakeholder intervention development group was constructed to ascertain whether agreement could be reached about the design, content, delivery, format and timing of the proposed occupational advice intervention. To facilitate this process a modified Delphi consensus process was employed (16). The initial research performed as part of the intervention mapping process provided the basis for this process by generating an initial list of statements for the Delphi consensus development.

For peer review only

Methods

Design of the modified Delphi study

A modified 3 round Delphi consensus process was used (19,20,21). The process was guided by the information gathered from research completed during the first phase of the OPAL project (16,17,18).

During the first phase of the OPAL project a number of intervention components emerged that were considered likely to be integral to the development of a successful occupational advice intervention (Table 1). Expanded versions of these components were used as the basis for initial statement development that could be explored during the Delphi process.

Delphi stakeholder recruitment

Five stakeholder groups were identified for inclusion in the modified Delphi process. The sampling strategy for each stakeholder group is outlined in Table 2, with participants chosen via a targeted approach to maximise patient, public and professional engagement. To ensure wide participation and the validity of the consensus process the process aimed to recruit a minimum of 5 individuals from each stakeholder group. A maximum limit of 15 individuals from any given stakeholder group was chosen to ensure one group's opinions did not overwhelm the opinions of others within the consensus process. As such, we aimed to have a minimum of 25 participants and a maximum of 75 participants for each round.

Development of Delphi statements

Prior to commencing, statements relating to the proposed **content, format, delivery, timing and measurement** of an occupational advice intervention were developed. Due to the breadth of statements developed and their inter-related nature, we adopted a step-wise approach to the presentation of individual statements to the Delphi group. Round 1 focussed on defining the **content** of the intervention in 2 sections. Section 1, focussed on passive content ('written' advice and information) and section 2 on active content (actions or processes for patients, employers and healthcare members to undertake). These statements were piloted by a small sample of surgeons, GPs and patients. Having first defined the content, we then used this information to refine the statements relating to the format, delivery, timing and measurement of this content presented in Round 2. In Round 2 statements were grouped under headings allowing exploration around specific themes. Round 3 was then used to clarify any areas of residual uncertainty from Rounds 1 and 2 and present the proposed occupational advice intervention back to the Delphi participants for final comments.

For each statement within the Delphi process participants were asked to rate the extent of agreement with individual statements about the importance of including specific elements in a occupational advice intervention, with possible options being: strongly agree; agree; disagree; strongly disagree; don't know. For a subset of statements in Round 1 they were also asked to rate the deliverability of the content or action alongside current healthcare provision. Therefore for some statements the participants were asked to provide two ratings one for 'importance' and one for 'deliverability'.

At the end of each section, there was a free txt box where participants could add suggestions relating to the intervention that could be evaluated in subsequent rounds. In rounds where statements from a previous Delphi round were being re-presented, these

For peer review only - http://bmjopen.bmj.com/site/about/guidelines.xhtml

were presented alongside controlled feedback with modal round one rating for these statements; the proportion of each response option selected by the other participants; and a reminder of the participant's own previous ratings.

Delivery of Delphi survey

The Delphi survey was delivered via email using an online web-based survey platform (31). Round 1 was delivered between 25 September 2017 and 13 October 2017, Round 2 between 22 November 2017 and 13 December 2017 and Round 3 between 01 June 2018 and 22 June 2018. The email included a covering letter to the participants and an electronic link to the questionnaires. Round 1 and 2 questionnaires required respondents to provide their initials and occupation. All Round 2 emails incorporated an overall report summarising the pooled responses from Round 1 survey and, where appropriate, the responses of each of the 5 stakeholder groups. In addition, for those participants who completed the Round 1 survey, an individualised report summarising their responses to the statements in Round 1 were included with the Round 2 survey to allow participants to reappraise their responses in view of the overall responses (32). Round 3 emails included 4 core documents from the developed occupational advice intervention (a summary of the intervention, occupational checklist, patient 'return to work' workbook and employer booklet) for participants to review and comment. Email reminders were sent to non-responders during the final week of the surveys.

Analysis of data

Descriptive analyses of the Delphi responses were undertaken by the OPAL study team. Results of each round were discussed with the wider OPAL study research team before the statements were agreed for subsequent rounds.

An a priori consensus threshold of 70% (strongly agree/agree or strongly disagree/sisagree) was agreed before statements were circulated (22). For statements that failed to reach consensus, further analysis was undertaken based on responses for each of the 5 stakeholder subgroups. The following rules were then employed to determine which statements were discarded and which were re-presented in the next round.

- If no or only one stakeholder group reached concordant consensus ($\geq$70% agreement or disagreement) then the statement would be withdrawn
- If two or more stakeholder groups reached concordant consensus ($\geq$70% agreement or disagreement) then the statement would be re-presented in a subsequent round.
- In the situation where one or more stakeholder groups reach 'agreement' and another group reach 'disagreement' the statement would be discussed by the OPAL investigator team and a decision on inclusion / exclusion of the statement would be made.

For statements that were rated for both importance and deliverability in Round 1, consensus was reached if the 70% threshold was achieved for both the importance and deliverability rating. Statements that reached consensus for one of the domains were analysed by stakeholder group as described above.

In Rounds 1 and 2 statements reaching consensus were ranked according to the distribution of responses to create a hierarchy of agreement.

In Round 3 the occupational advice intervention and associated documents were circulated for comment. Descriptive open feedback from participants to these documents were recorded.

Ethics Approval-

This study was reviewed and given favourable opinion by the East Midlands - Derby Research Ethics Committee (16/EM/0341) and approved by the Health Research Authority in UK.

For peer review only

Results

Round 1:

Responses were received from 43 of the 66 participants (65%) including 14 patients, 8 surgeons, 6 GPs, 11 allied health professionals and nurses, and 4 employers. In section 1 ('written' advice and information), consensus was reached for 26 of 32 statements (81%). Of the remaining 6 statements, 5 reached consensus for 2 or more stakeholder groups and were therefore taken forward to Round 2 and one statement was discarded. Section 1 statements reaching consensus and ranked based on the strength of consensus are listed in Table 3.

In section 2 (actions or processes for patients, employers and healthcare members to undertake), participants were asked to rate both the importance and deliverability of each statement. Of the 32 components presented only 10 (31%) reached consensus for both importance and deliverability (Table 4). Of the remaining 22 statements, 14 reached consensus for importance but not deliverability, 2 reached consensus for deliverability but not importance and 6 did not reach consensus for either. Of these statements 7 reached consensus for both importance and deliverability for 2 or more stakeholder groups and were therefore taken forward to Round 2 and 15 statements were discarded.

Round 2:

Responses were received from 26 of the 66 participants (39%) including 8 patients, 7 surgeons, 3 GPs, 6 allied health professionals and nurses, and 2 employers.

Twelve questions carried forward from Round 1 plus one additional question generated from the free text comments were presented to the participants. Of these 10 reached consensus based on their potential importance within the proposed occupational advice intervention.

A further 81 statements grouped into 13 categories were then rated. This allowed the team to explore different approaches to a given problem. For example the first category asked participants to rate a set of 5 statements relating to which healthcare team member should have responsibility for delivery and co-ordination of the occupational advice intervention. If at least one or more statements in a given category reached consensus this was taken as representative of the Delphi group's position relating to the given category and the remaining statements were discarded. Overall 49 statements (60%) reached consensus (44 agreement and 5 disagreement), at least one statement in every category reached consensus (Appendix Table 1).

The Occupational advice intervention:

Based on the evidence gathered throughout the OPAL study and consolidated through Delphi rounds 1 and 2 the occupational advice intervention was further developed and finalised.

The intervention was designed to support patients throughout their surgical pathway, starting during their initial outpatient appointment and continuing until 16 weeks after surgery. It had a number of key themes that linked to performance objectives for patients and staff and was supported by a range of patient and staff resources (to support delivery

For peer review only - http://bmjopen.bmj.com/site/about/guidelines.xhtml

and measurement of the intervention). A simplified schematic of the OPAL 'return to work' intervention is presented in figure 1.

Round 3:

In Round 3, the finalised occupational advice intervention along with selected patient and staff materials were circulated to 65 of the 66 Delphi participants for comment (one patient withdrew). Responses were received from 11 participants comprising a constructive appraisal of the intervention from 9, as well as highlighting typographical and formatting issues. The feedback was positive in all cases (Appendix Table 2).

For peer review only

Discussion

The Delphi consensus methodology was used to underpin the development of an evidence-based, theory-driven occupational advice intervention to assist patients returning to work after elective hip and knee replacement. It enabled the OPAL study team to rationalise the content, format, delivery and timing of the intervention and clarified areas of uncertainty related to the intervention that had arisen during the earlier stages of the research. Response to the developed intervention during the third round of the Delphi process was positive, validating the use of the Delphi process to support intervention development.

Prior to the Delphi process, the OPAL study had already completed a number of complementary research phases to enable the OPAL team understand the current evidence, stakeholder and patient perspectives, and current practice relating to return to work after hip and knee replacement (16,17,18). Through the intervention mapping framework this is information generated a range of components for our intervention. The Delphi methodology was then used to 'refine' the intervention and reach consensus on the final design. This is similar to the modified Delphi approach used by Vonk Noordegraaf el al to develop a return to work intervention for gynaecological surgery, as it used existing evidence as the basis for the process but sought to bridge gaps and clarify uncertainty within this evidence (23). However one limitation of this approach is that it may inadvertently narrow the focus of the intervention with only components deemed important by the research team included. There is a risk that potentially useful intervention components that may have been of interest to the stakeholder groups were not included as the starting position was pre-defined. However, the approach used is not unusual and is similar to the approaches used by others (20, 33). Furthermore, given the breadth of work completed earlier in the OPAL study and the design of the modified Delphi survey allowing participants to suggest new intervention components within each round, this is unlikely to have had a negative impact.

Broad stakeholder involvement helped the research team ensure the final intervention was acceptable to all groups, increasing the chances of success when implemented and delivered. Unfortunately despite good initial engagement, the response rates reduced as the process progressed. This is a common finding during Delphi processes (20) and was perhaps related to the larger sample size involved and extended period of the process with a 6 week gap between Round 1 and 2 and a 6 month gap between Rounds 2 and 3. The gap between Round 2 and 3 was necessary as the intervention needed to be finalised with associated materials being developed during this period. Other contributing factors may include the increasing length of the Delphi questionnaires with each round and the volume of materials that needed to be reviewed in Round 3.

While the low response rate in Round 3 may be a concern, the purpose of this round was to circulate and draw comment regarding the final intervention rather than reach consensus on specific points. With 11 respondents, including at least one member from each stakeholder group, this seems valid as Delphi process relies more on the group dynamics even with reaching consensus rather than their statistical power and a lower limit of 10 participants is often considered sufficient for a Delphi panel (34,35). During the process there was a notable drop off in employer respondents. In total 12 employers initially expressed an interest in participating, however only four responded in round 1, two in round 2 and one in round 3. It is often difficult to engage employers in research (24) and despite using a number of complementary strategies (17) we were unable to maintain engagement. However, as the intervention was designed to be delivered in secondary care rather than in the workplace this potentially did not significantly influence the nature of the final intervention.

For peer review only

The modified Delphi methodology employed in this study resolved uncertainties about a number of intervention components. However, there were a few areas where the consensus process was limited. Two key areas that stakeholders felt were important were 1) the provision of additional pre and post-operative physiotherapy/occupational therapy (over and above standard care) in which return to work issues could be addressed and 2) the identification of 'high risk' patients that should be provided with additional help and support. Yet, these positions conflicted with other information gathered from the Delphi participants and the evidence from OPAL phase 1. Essentially firstly, our cohort study failed to identify a 'high risk' population and the current literature describing predictors of return to work after hip and knee replacement was limited (25-30). This meant we were not able to confidently identify a 'high risk' group in need of a more intensive targeted intervention. Secondly, there was concern about the cost, time and logistics associated with the implementation of a resource intensive intervention requiring additional patient interactions. The survey of practice and stakeholder / patient interviews demonstrated that services varied significantly in their structure and the resources available (18). To be successful it was agreed that the intervention should supplement rather than replace existing pathways of care and should, where possible, use existing staff and adapt current working. Comments from Delphi participants, the OPAL research team and the study steering committee had similarly raised concerns about the implementation and sustainability of an intervention requiring significant additional resources. The OPAL research team therefore felt that, despite this component reaching consensus, it was prudent to pursue a less intensive model to improve implementation of the final intervention.

In conclusion, a modified Delphi consensus process employed within a wider intervention development project facilitated the development of the OPAL occupational advice intervention. Consensus was reached for a range of intervention components that allowed the content, format, delivery and timing of the intervention to be finalised. The intervention developed and the materials created to support its delivery were well received by the Delphi group. The effectiveness and cost-effectiveness of the developed intervention will require evaluation in a randomised controlled trial.

For peer review only

OPAL Collaborators
The following researchers contributed to the concept, design, delivery and analysis of the OPAL study:
Sayeed Khan (Make UK, the Manufacturers Organisation), Louise Thomson (Faculty of Medicine and Health Sciences, University of Nottingham), Catherine Hewitt and Catriona McDaid (York Trials Unit, University of York), Iain McNamara (Norfolk and Norwich University Hospital NHS Foundation Trust), David McDonald (University of Stirling), Judith Fitch (British Orthopaedic Association Patient Liaison Group), Gerry Richardson (Centre for Health Economics, University of York)

The authors acknowledge the contributions of other members of the wider OPAL study team: Reece Walker and Amanda Goodman (South Tees Hospitals NHS Foundation Trust); Sarah Ronaldson and Elizabeth Coleman (York University); Dr Fiona Nouri and Dr Melanie Narayanasamy (University of Nottingham).

The OPAL investigators acknowledge the support of the British Orthopaedic Association Orthopaedic Surgery Research Centre (BOSRC) which supports the development of surgical trials.

Funding acknowledgement
This project was funded by the National Institute for Health Research Health Technology Assessment (HTA) programme (project number 15/28/02). Further information available at https://www.journalslibrary.nihr.ac.uk/programmes/hta/152802/#/
The funding body have not been involved in the design of the study, data collection, data analysis and data interpretation. They have not been involved in the writing of this manuscript.

Department of Health and Social Care disclaimer
This report presents independent research commissioned by the National Institute for Health Research (NIHR). The views and opinions expressed by the authors in this publication are those of the authors and do not necessarily reflect those of the National Health Service, the National Institute for Health Research, Medical Research Council, Central Commissioning Facility, NIHR Evaluations, Trials and Studies Coordinating Centre, the Health Technology Assessment programme or the Department of Health.

Competing interests
The authors declare they have no competing interests

Author Contributions
- Paul Baker: If the Chief Investigator for the OPAL study. Contributed to the conception of the study, the design of the work; the acquisition, analysis, or interpretation of data for the work; drafted the manuscript.
- Lucksy Kottam: Contributed to the design of the work; the acquisition, analysis, or interpretation of data for the work; drafted the manuscript.
- Carol Coole: Contributed to the conception of the study, the design of the work; the interpretation of data for the work; editted the manuscript.
- Avril Drummond: Contributed to the conception of the study, the design of the work; the interpretation of data for the work; editted the manuscript.
- Catriona McDaid: Contributed to the conception of the study, the design of the work; the interpretation of data for the work; editted the manuscript.

For peer review only

- Amar Rangan: Contributed to the design of the work; the acquisition, analysis, or interpretation of data for the work; drafted the manuscript.

Patient and Public Involvement

The OPAL research project was developed in collaboration with members of the British Orthopaedic Association (BOA) Patient Liaison Group (PLG). A patient co-applicant from the BOA PLG was involved in the development of the research question and defining the outcome measures used within the wider OPAL study. Patients were involved in the design of the study from inception of the project, through protocol development, study delivery and project dissemination. These included patients from the BOA PLG, the National Joint Registry patient group and patient and public groups affiliated with the sponsor site.

For peer review only

References

1. Bieleman HJ, Bierma-Zeinstra SM, Oosterveld FG et al. The effect of osteoarthritis of the hip or knee on work participation. J Rheumatol 2011;38:1835-43
2. Shariff B, Garner R, Sanmartin C et al. Risk of work loss due to illness or disability in patients with osteoarthritis: a population based cohort study. Rheumatology 2016;55:861-8.
3. Bhattacharrya A. Costs of musculoskeletal disorders (MSDs) in the United States. International Journal of Industrial Ergonomics (2014); 44(3):448-454.
4. HSE lower limb disorders (2018) Accessed at http://www.hse.gov.uk/msd/lld/employees.htm
5. HSE Cost of workplace injury and Ill health (2017) Accessed at http://www.hse.gov.uk/statistics/cost.htm
6. Gignac M, Badley E, Lacaille D, Cott C, Adam P, Anis A. Managing arthritis and employment: Making arthritis-related work changes as a means of adaptation. Arthristis and Rheumatism 2004;51(6);909-916
7. O'Brien G, Feather N. The relative effects of unemployment and quality of employment on the affect, work values and personal control of adolescents. J Occup Psychol 1990;63:151-65.
8. Waddell G, Burton AK. Is work good for your health and well-being? London UK: The stationary office 2006.
9. Jones CA, Voaklander DC, Johnston DW, Suarez-Almazor ME. Health related quality of life outcomes after total hip and knee arthroplasties in a community based population. Journal of Rheumatology 2000;27:1745–52.
10. Ethgen O, Bruyere O, Richy F, Dardennes C, Reginster JY. Health-related quality of life in total hip and total knee arthroplasty. A qualitative and systematic review of the literature. J Bone Joint Surg Am 2004;86-A:963e74.
11. Fitzgerald JD, Orav EJ, Lee TH, Marcantonio ER, Poss R, Goldman L, Mangione CM. Patient quality of life during the 12 months following joint replacement surgery. Arthritis and rheumatism 2004;51(1):100-9.
12. Bachmeier CJ, March LM, Cross MJ, Lapsley HM, Tribe KL, Courtenay BG, Brooks PM. A comparison of outcomes in osteoarthritis patients undergoing total hip and knee replacement surgery. Osteoarthritis Cartilage 2001;9:137–46.
13. NJR Knee data (2018) Accessed at http://www.njrreports.org.uk/hips-primary-procedures-patient-characteristics/H05v1NJR?reportid=351A3B2E-F983-4D53-BC1E-D9A90DC93AAA&defaults=DC__Reporting_Period__Date_Range="MAX",J__Filter__Calendar_Year="MAX",H__Filter__Joint="Hip"
14. NJR Knee data (2018) Accessed at http://www.njrreports.org.uk/knees-primary-procedures-patient-characteristics/K07v1NJR?reportid=351A3B2E-F983-4D53-BC1E-D9A90DC93AAA&defaults=DC__Reporting_Period__Date_Range="MAX",J__Filter__Calendar_Year="MAX",H__Filter__Joint="Knee"
15. NJR 15th Annual Report; National Joint Registry for England, Wales, Northern Ireland and the Isle of Man Surgical data to 31 December 2017 (2018)
16. Baker P, Coole C, Drummond A, McDaid C, Khan S, Thomson L, Hewitt C, McNamara I, McDonald D, Fitch J, Rangan A. Protocol paper: Occupational advice for Patients undergoing Arthroplasty of the Lower limb (OPAL). BMC Health services Research 2018 18:504
17. Coole C, Drummond A, Khan S, Baker P, Nouri F, Narayanasami M. Engaging employers in research: What strategies to consider, what works and what does not work Occupational Medicine, Volume 68, Issue 4, 23 May 2018, Pages 282–285

For peer review only

18. Nouri F, Coole C, Narayanasamy M, Baker P, Khan S, Drummond A. Managing employees undergoing total hip and knee replacement; experiences of workplace representatives. Journal of occupational rehabilitation ():1-11
19. Jones J and Hunter D. Consensus methods for medical and health services research. *BMJ* 1995; 311: 376-311.
20. Sinha IP, Smyth RL, Williamson PR. Using the Delphi technique to determine which outcomes to measure in clinical trials: recommendations for the future based on a systematic review of existing studies. PLoS. Med. 2011;8(1):e1000393.
21. Murphy MK, Black NA, Lamping DL, McKee CM, Sanderson CF, Askham J, et al. Consensus development methods, and their use in clinical guideline development. Health Technol Assess. 1998;2(3):i-iv, 1-88.
22. Keeney S, McKenna H and Hasson F. *The Delphi Technique in nursing and health research.* 1st edition. Chichester: John Wiley and Sons Ltd, 2011.
23. Vonk Noordegraaf A, Huirne J, Brolmann H, van Mechelen W, Anema J. Multidiciplinary convalescence recommendations after gynaecological surgery: a modified Delphi method among experts. BJOG 2011;118:1557-1567.
24. Lysaght R, Kranenburg R, Armstrong C, Krupa. Participant recruitment for studies on disability and work: challenges and solutions. *J Occup Rehabil* 2016; 26: 125-140.
25. Tilbury C, Schaasberg W, Plevier JW, et al. Return to work after total hip and knee arthroplasty: a systematic review. *Rheumatology (Oxford, England)* 2014;53(3):512-25. doi: 10.1093/rheumatology/ket389 [published Online First: 2013/11/26]
26. Kuijer PPF, de Beer MJP, Houdijk JHP, et al. Beneficial and limiting factors affecting return to work after total knee and hip arthroplasty: a systematic review. *Journal of Occupational Rehabilitation* 2009;19(4):375-81. doi: 10.1007/s10926-009-9192-1
27. Sankar A, Davis AM, Palaganas MP, et al. Return to work and workplace activity limitations following total hip or knee replacement. *Osteoarthritis and cartilage* 2013;21(10):1485-93. doi: 10.1016/j.joca.2013.06.005 [published Online First: 2013/06/19]
28. Tilbury C, Leichtenberg CS, Tordoir RL, et al. Return to work after total hip and knee arthroplasty: results from a clinical study. *Rheumatology international* 2015;35(12):2059-67. doi: 10.1007/s00296-015-3311-4 [published Online First: 2015/06/30]
29. Leichtenberg CS, Tilbury C, Kuijer P, et al. Determinants of return to work 12 months after total hip and knee arthroplasty. *Annals of the Royal College of Surgeons of England* 2016;98(6):387-95. doi: 10.1308/rcsann.2016.0158 [published Online First: 2016/05/04]
30. Scott CEH, Turnbull GS, MacDonald D, et al. Activity levels and return to work following total knee arthroplasty in patients under 65 years of age. *The bone & joint journal* 2017;99-b(8):1037-46
31. Survey Monkey Inc., San Mateo, California, USA. www.surveymonkey.com
32. Christina Vogel, Stephen Zwolinsky, Claire Griffiths, Matthew Hobbs, Emily Henderson & Emma Wilkins. A Delphi study to build consensus on the definition and use of big data in obesity research. 17 January 2019, Epidemiology and population health.
33. Slade SC, Dionne CE, Underwood M, et al Standardised method for reporting exercise programmes: protocol for a modified Delphi study BMJ Open 2014;4:e006682. doi: 10.1136/bmjopen-2014-006682
34. Chitu Okoli, Suzanne D. Pawlowski, The Delphi method as a research tool: an example, design considerations and applications, Information & Management, Volume 42, Issue 1, 2004, Pages 15-29, ISSN 0378-7206.

35. Akins, R., Tolson, H., & Cole, B. (2005). Stability of response characteristics of a Delphi panel: Application of bootstrap data expansion. BMC Medical Research Methodology, 5(37).

For peer review only

Tables

Key intervention components identified during OPAL phase 1
• Education and advice<br>• Vocational counselling and guidance<br>• Physical therapy and exercises<br>• Work simulation/work hardening and job accommodation<br>• Contact with employer/workplace visits<br>• Multidisciplinary team involvement

Table 1: Key intervention components identified during OPAL phase 1

For peer review only

Stakeholder group	Requirement for inclusion	Participants recruited via:
Patients	Experience of returning to work after hip or knee replacement in the previous 12 months	• National Joint Registry patient network<br>• British Orthopaedic Association patient group<br>• Patient participants from OPAL phase 1
Employers and occupation health services	Experience of managing an employee returning to work after hip or knee replacement in the previous 12 months	• Federation of Small Businesses<br>• EEF – The manufacturers organisation<br>• Confederation of British Industry<br>• Trade Union Congress<br>• Department for Work and Pensions<br>• The Fit for Work Service<br>• The Work Foundation<br>• The Society of Occupational Medicine<br>• Institution of Occupational Safety & Health<br>• Society of Occupational Health Nurses
Orthopaedic Surgeons	Surgeons undertaking a minimum of 20 hip or knee replacements per year	• British Hip Society<br>• British Association for Surgeon of the Knee<br>• British Orthopaedic Association<br>• National Joint Registry
Allied Health Professionals (AHPs – Physiotherapists and Occupational therapists) and nurses	AHPs actively involved in the assessment and / or management of patients undergoing hip or knee replacement	• Association of Chartered *Physiotherapists* in *Occupational* Health and Ergonomics<br>• Chartered Society of Physiotherapy<br>• Occupational therapy networks e.g. College of Occupational Therapists Specialist Sections in Work and Trauma & Orthopaedics<br>• Royal College of nursing
General Practitioners	Experience of managing a patient returning to work after hip or knee replacement in the previous 12 months	• Local Medical Committees<br>• Royal College of General Practitioners<br>• Local Clinical Commissioning Groups

Table 2: Sampling strategy used to identify Delphi members

For peer review only

Is it important that an occupational advice intervention commenced prior to hip or knee replacement includes the following:	Agreement (%)
Q9. Information about exercises and rehabilitation following surgery	100%
Q13. Information about returning to driving	100%
Q3. A broad overview written for all stakeholders, of what to expect following surgery (rates and timing of expected recovery)	98%
Q15. Information about managing pain, types of analgesia and side effects	98%
Q5. Information about post-operative precautions, restrictions and activities to avoid following surgery	95%
Q18. Information about symptom management in relation to return to work and specific occupations e.g. expected levels of fatigue, pain, swelling	95%
Q12. Tips and tricks to help the patient manage around their home with day to day activities immediately following surgery	95%
Q10. Information regarding post-operative complications and their management	95%
Q14. Signposting to DVLA guidance	95%
Q23. Information for the patient about who to ask if they are having a problem returning to work	93%
Q4. Information about expected level of function at different time - points following surgery	88%
Q29. Advices about adaptions to working patterns to assist return including the use of phased returns, modified hours and altered work schedules	88%
Q21. Information and resources to support self-advocacy and empowerment	88%
Q20. Information about when it might be appropriate for patients and employers to access occupational health services	88%
Q19. Information for patients and employers about how to access occupational health services	88%
Q28. A list of potential workplace modifications, aids and adjustments that could be used to assist with return to work, with examples	84%
Q27. Information for the patients about how to ask for help at work from their employer and colleagues	84%
Q31. Guidance on how to set an appropriate provisional return to work date based on the date and type of surgery	81%
Q16. Guidance for orthopaedic care teams and G.Ps on how to use and prescribe a fit note	81%
Q24. Signposts to national and local support services e.g. Fit4Work, Citizens advices, ACAS	81%
Q11. Information about how having surgery may impact on social relationships	81%
Q32. Advice about how psychosocial and emotional factor influence return to work	79%
Q6. Information about how long the hip and knee replacement prostheses will last	77%
Q17. Examples of the correct use of fit notes	77%
Q30. A list of potential return to work barriers for patients and employers to consider	74%
Q8. Information about managing more than one joint replacement in close succession	72%

Table 3: Statements descriptions reaching consensus for section 1 (Ordered by % of respondents that strongly agreed or agreed)

For peer review only

**How important / deliverable do you believe the following components are if an occupational advice intervention commencing prior to hip or knee replacement were to be developed**	**Agreement (%)**	**Agreement (%)**
**10 statements reaching consensus for both IMPORTANCE and DELIVERABILITY**		
Q37. A post-operative mechanism for the identification of patients that are not progressing toward return to work as planned	95%	71%
Q52. Guidance for health services defining 'best practice' for patients returning to work after hip and knee replacement surgery	93%	82%
Q45. Training for members of the hospital orthopaedic care team to increase awareness about return to work issues	88%	82%
Q42. Interaction between the healthcare team and patient by phone, email or 'on-line' so that members of the care team can monitor progress and help the patient use the advice and information provided	88%	70%
Q64. Guidance on when in the return to work process patients can safely be discharged back to primary care for continued management of their return to work	86%	80%
Q36. A mechanism for pre-operative identification of patients at 'high risk' of prolonged sickness absence following surgery	86%	74%
Q51. Routine pre-operative therapy assessment during which a return to work plan is developed between the patients and the hospital orthopaedic care team	84%	80%
Q40. A separate intervention for hip and knee replacement patients that are not progressing towards return to work as planned	84%	79%
Q62. A process by which work status can be included in referral information for all patients referred from primary care into secondary care for consideration of hip or knee replacement	79%	79%
Q57. Information from patients that have experienced the process of returning to work after hip or knee replacement within the pre-operative education process	76%	73%

Table 4: Statements descriptions reaching consensus for section 2 (Ordered by % of respondents that strongly agreed or agreed)

For peer review only

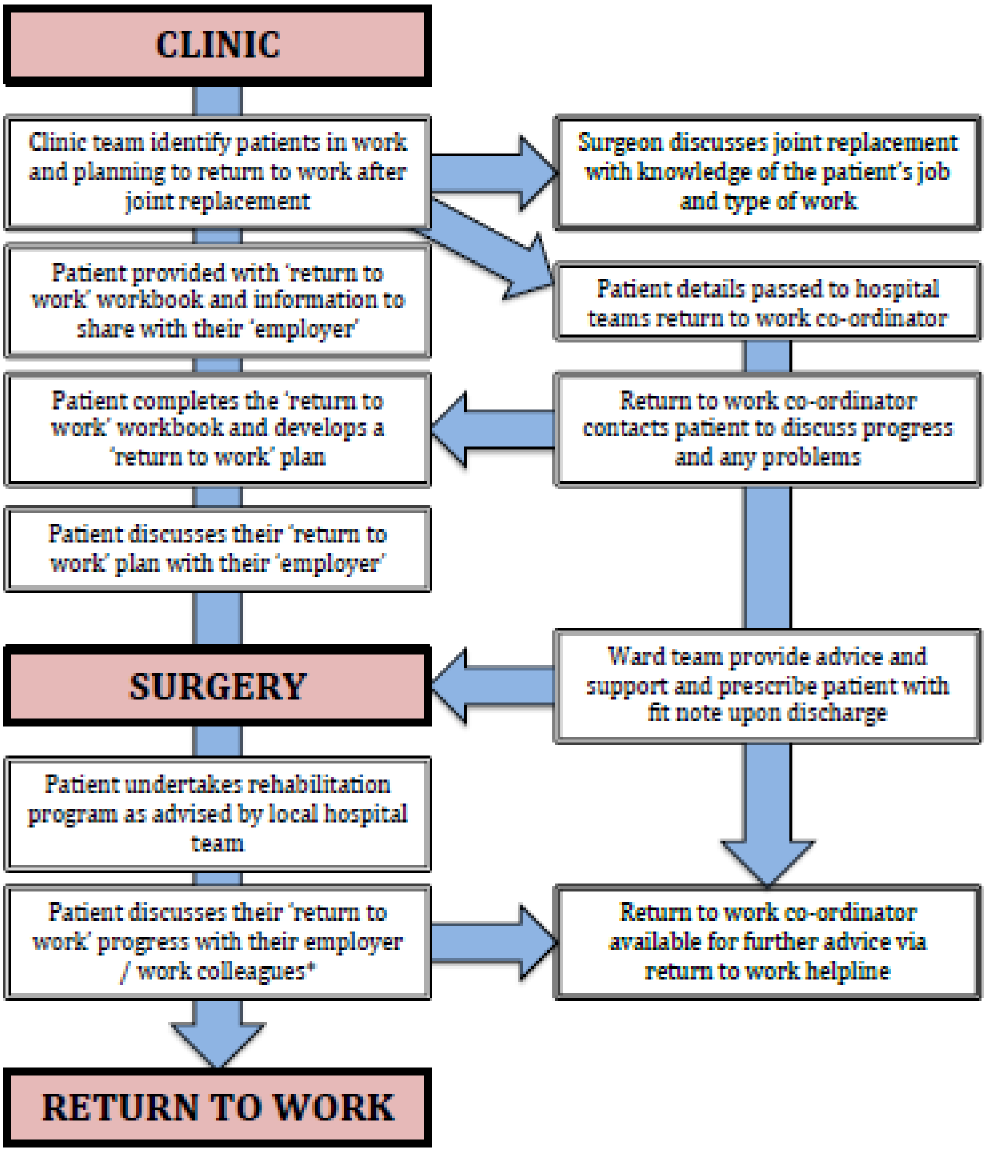

Figure 1: Simplified schematic of the OPAL 'return to work' intervention

Appendices

	Category	Statements reaching consensus
1	Responsibility for delivery and co-ordination of the return to work intervention	Individual orthopaedic surgeons should be responsible for co-ordinating the return to work intervention for their own patients (88% disagreement)
2	Pre-operative identification of patients at 'higher risk' of prolonged sickness absence following surgery that may require additional individualised help and support	A face-to-face assessment with a staff member trained in the return to work intervention should be used to identify patients at 'higher risk' of prolonged sickness absence following surgery (80% agreement)
3	Pre-operative needs assessment	Hospital physiotherapy teams should see all patients intending to return to work prior to their surgery (80% agreement)<br><br>Hospital occupational therapy teams should see all 'higher risk' patients intending to return to work prior to their surgery (88% agreement)<br><br>Hospital physiotherapy team should see all 'higher risk' patients intending to return to work prior to their surgery (80% agreement)<br><br>Development of a return to work plan should be a routine part of the pre-assessment process for all patients undergoing hip and knee replacement (80% agreement)<br><br>Development of a return to work plan should be a routine part of the pre-assessment process for all 'higher risk' patients undergoing hip and knee replacements (96% agreement)<br><br>Pre-operative education sessions conducted by the hospital orthopaedic team should routinely include information about returning to work after surgery (88% agreement)<br><br>Patients should be encouraged to have a meeting with their employer prior to surgery to discuss their recovery and plans for returning to work (100% agreement)
4	Post-operative identification of patients at risk of an extended period off work after surgery	All return to work patients should be offered a routine physiotherapy review after discharge from hospital to help monitor their progress and identify those patients at risk of an extended period off work after surgery (84% agreement)<br><br>All return to work patients should be contacted by a member of the therapy team after discharge to help monitor their progress and identify those patients at risk of an extended period off work after surgery (92% agreement)<br><br>The hospital should provide a means of contact (phone, email etc) through which patients in need of additional help and support can contact the hospital orthopaedic team (92% agreement)<br><br>Patients should be encouraged to have a meeting with their employer in the 4 weeks following surgery to discuss their recovery and plan for return to work (72% agreement)
5	Additional care for Patients identified as 'higher risk' of an extended period off work after surgery	Additional post-operative physiotherapy review with a physiotherapist until specific work related rehabilitation targets have been achieved (92% agreement)<br><br>Additional post-operative occupational therapy review (76% agreement)<br><br>Ongoing therapy involvement until 'return to work' has been achieved

For peer review only

		(76% agreement)<br><br>Access to additional procedure specific information and advice (92% agreement)<br><br>Referral to the Fit4Work service (84% agreement)
6	Scope of training for staff	All members of the hospital orthopaedic team involved in the treatment of hip and knee replacement patients should receive training regarding the return to work intervention (76% agreement)<br><br>Specific staff members with responsibility for delivering 'return to work' advice should receive training regarding the return to work intervention (96% agreement)<br><br>All members of the therapy teams treating hip and knee replacement patients should receive training regarding the return to work intervention (88% agreement)<br><br>Training regarding the return to work intervention should be offered to local G.P groups (72% agreement)
7	Communicating occupational status and progress between stakeholders	The details of the patient's occupational status should be recorded in the primary care referral letter (96% agreement)<br><br>Details of the patient's occupational status should be confirmed and recorded in all interactions with secondary care (e.g. pre-operative outpatient appointments, discharge summaries post-operative follow-up appointment letters) (84% agreement)<br><br>Any information developed with the healthcare team as part of a patient return to work plan should be made available to the G.P (96% agreement)<br><br>Any information developed with the healthcare team as part of a patient return to work plan should be made available to the patient's employer and/or occupational health provider with their consent (84% agreement)<br><br>It is the patients and not the healthcare teams responsibility to communicate with their employer about their return to work (76% agreement)<br><br>All post-operative assessments by members of the therapy team should generate a statement about the patient's current functional status and estimated timeframe to return to work that is given to the patient and forwarded to their G.P (84% agreement)
8	Fit Notes	Fit notes should be completed in accordance with Department for Work and Pensions Fit Note Guidance (96% agreement)<br><br>Hospital orthopaedic departments should actively ask each and every patient if they require a fit note prior to discharge (96% agreement)<br><br>To discourage extended periods off work patients should be issued with a basic fit note excusing them from work for 2 weeks. Further fit notes can be issued based on progress towards recovery and access to phased return or amended duties and hours (72% disagreement)<br><br>G.Ps should be responsible for administering subsequent fit notes based on progress reports from the hospital orthopaedic team (76% agreement)
9	Format and delivery of patient information	All of the information and advice components relevant to patients should be included within a written patient booklet that is given to all patients. Information for other stakeholders will be delivered separately (76% agreement)<br><br>Only the most important (based on the ranking listed previously) information and advice components relevant to patients should be

For peer review only

		included within a written patient booklet that is given to all patients. Additional information could be made available via a website (80% agreement)<br><br>The patient booklet should include a section within which the patient and other stakeholders can record progress and feedback. This information would be held by the patient and could be shared with their permission (80% agreement)<br><br>The patient booklet should include a section within which the patient can record the outcome of any meetings and with their employer and/or occupational health team. This information would be held by the patient and could be shared with their permission (92% agreement)<br><br>Patients with previous experience of returning to work after joint replacement should be involved in the drafting of any patient facing materials (92% agreement)<br><br>The occupational advice intervention should include the ability for local hospital department to upload their own policies and procedures relating to post-operative recovery and return to work (92% agreement)<br><br>Primary Care Health professional and support staff should be able to direct potential surgical candidates to return to work information and advice prior to referral (88% agreement)
10	When should the intervention commence?	The intervention should commence in secondary care after the patient has had their operation but prior to discharge (88% disagreement)
11	Defining return to work	A patient has 'returned to work' only when they return to the same job (usual hours and duties) and activities outside of work they were doing prior to surgery (72% disagreement)
12	The aim of the intervention	The return to work interventions primary aim should be to return the patient to their pre-operative work role / level of occupational performance. (76% agreement)
13	Measuring return to work	The measurement of return to work should be graded based on specific work milestones for example return to place of work, return to normal hours, return to normal workplace activities (92% agreement)<br><br>The measurement of return to work should be based on resumption of specific work related activities (e.g. climbing, lifting, manual tasks) based on a list of pre-operative workplace activities (72% agreement)<br><br>The measurement of return to work is a binary measure – the patient has either returned to work or they have not (72% disagreement).<br><br>The rates of further sick leave in the 6 months after the patient has returned to work once is an important outcome measure (80% agreement)<br><br>The rates (type and duration) of any occupational adjustments implemented by the employer is an important outcome measure (84% agreement)<br><br>Asking patients about how they feel about their return to work is important outcome measure (96% agreement)<br><br>An assessment of whether or not the patient's preoperative occupational expectations following surgery have been met is an important outcome measure (92% agreement)

Appendix Table 1: Statements descriptions reaching consensus for Round 2

For peer review only

Stakeholder group	Comments
Surgeon	It all looks very good. Very thorough. A lot for the patient to read but I guess there is a lot for them to go through. The employer booklet is good
BASK member	Having been slightly sceptical about some of the outcomes from the project I think you have done a great job, it's a superb resource.
BASK member	At first glance they [the patient and employer workbooks] appear long and complicated. However, on reflection, the patients who want to return to work will read them religiously, and those employers who want to support their staff will do so as it gives detailed and practical advice.
Surgeon	The documents appear very professional with lots of information. Excellent job. My concern is that they are large and may be seen as a lot of extra work for already overworked staff. Looks like it will require dedicated staff ?
Research physiotherapist	Well done team…this looks a serious amount of work and it is great seeing it all together
RCGP lead	I fully support the pathway that you have designed and the accompanying materials are superb - we just have to hope that the system is adequately resourced. I have been discussing the problem of return to work with Lord Luce - the latter is pain management's biggest supporter in the House of Lords. He has a particular interest in pain and work and indeed chaired a focus group to feed back on the governments green paper on work.
Employer: Head of Safety, Health and Quality	Thank you for the documents they are very interesting and informative. XXXXX are already following the majority of the information for an employee returning to work after a Hip or Knee replacement. In our case the employee would return to work on a 'phased return' and 'restricted duties', we will also at this time adjust the start and working hours and if applicable, the work load. The checklist would be beneficial as it would probably provide more time to make the adjustments as the patient would read it on discharge and probably not leave it until they were due start back to work.
NJR Patient Liaison Group member	I have reviewed the materials and think they are excellent.<br><br>I am hopeful that the specified commitment required to support those of working age return to work after surgery is available. The level and range of contact and time required is considerable. Without that commitment this programme will not be so successful. I note on page 5 there is a suggestion to share the workbook knowledge with the patient's GP. In fact the discharged patient may have a lot of contact post surgery with the GP for purpose of prescription pain relief and signing of 'fit notes' so I consider it vital to involve/inform the GP team about the Return To Work intervention programme at the earliest stage.<br><br>The layout and explanation is clear. However, there is a lot to take in and consider so the patient needs to receive RTW booklet asap. This is indicated in the diagrammatic guide to the process on page 8. The design with checklists and tickboxes makes the patient face the practical issues and really think about any obstacles on the journey back to work. This also has the effect of 'setting the agenda' for patients to move away from an entirely 'medical" model of surgery to fix a problem - to more about enabling them to continue to being active and independent.<br><br>Some patients will need more assistance and guidance than others as there is a lot of text. EG. Page 13 is suggesting that 10 weeks is a average target time for return to work , and that type of work had no real impact. These are key messages and could be at the top of the page followed by evidence.<br><br>I am aware of Dame Carol Black's review of the health of the working age population and its impact on the economy. This initiative will be valuable and could be rolled out for wider application. I think the OPAL pathway to return to work identifies the vital role of taking the patient past the immediate recovery from surgery to the place where shared decision making, timely support and understanding gets them back into their life.
Patient / Ambassador for Global alliance for MSK Health of the bone and joint decade	I think the work that the OPAL Team have worked wonders. The patient and employer booklets are first class... I'd love to see the slides in due course, please.<br>What is the time line to rolling out the work? When can I share these data with my colleagues at the BOA's Patient Liaison group

Appendix Table 2: Comments received as part of Delphi Round 3

For peer review only

BMJ Open

Development of an occupational advice intervention for patients undergoing elective hip and knee replacement: A Delphi study

Journal:	*BMJ Open*
Manuscript ID	bmjopen-2019-036191.R1
Article Type:	Original research
Date Submitted by the Author:	15-Apr-2020
Complete List of Authors:	Baker, Paul; South Tees Hospitals NHS Foundation Trust, Department of Orthopaedics ; University of York,<br>Kottam, Lucksy; South Tees Hospitals NHS Foundation Trust, Department of Orthopaedics<br>Coole, Carol; University of Nottingham, 3. School of Health Sciences, Faculty of Medicine & Health Sciences<br>Drummond, Avril; University of Nottingham, Div of Rehabilitation and Ageing<br>McDaid, Catriona; University of York, York Trials Unit<br>Rangan, Amar; South Tees Hospitals NHS Foundation Trust, Trauma and Orthopaedics; University of York, Department of Health Sciences
**Primary Subject Heading**:	Occupational and environmental medicine
Secondary Subject Heading:	Health policy, Health services research, Patient-centred medicine, Surgery
Keywords:	OCCUPATIONAL & INDUSTRIAL MEDICINE, Adult orthopaedics < ORTHOPAEDIC & TRAUMA SURGERY, Hip < ORTHOPAEDIC & TRAUMA SURGERY, Knee < ORTHOPAEDIC & TRAUMA SURGERY, Musculoskeletal disorders < ORTHOPAEDIC & TRAUMA SURGERY

SCHOLARONE™
Manuscripts

I, the Submitting Author has the right to grant and does grant on behalf of all authors of the Work (as defined in the below author licence), an exclusive licence and/or a non-exclusive licence for contributions from authors who are: i) UK Crown employees; ii) where BMJ has agreed a CC-BY licence shall apply, and/or iii) in accordance with the terms applicable for US Federal Government officers or employees acting as part of their official duties; on a worldwide, perpetual, irrevocable, royalty-free basis to BMJ Publishing Group Ltd ("BMJ") its licensees and where the relevant Journal is co-owned by BMJ to the co-owners of the Journal, to publish the Work in this journal and any other BMJ products and to exploit all rights, as set out in our licence.

The Submitting Author accepts and understands that any supply made under these terms is made by BMJ to the Submitting Author unless you are acting as an employee on behalf of your employer or a postgraduate student of an affiliated institution which is paying any applicable article publishing charge ("APC") for Open Access articles. Where the Submitting Author wishes to make the Work available on an Open Access basis (and intends to pay the relevant APC), the terms of reuse of such Open Access shall be governed by a Creative Commons licence – details of these licences and which Creative Commons licence will apply to this Work are set out in our licence referred to above.

Other than as permitted in any relevant BMJ Author's Self Archiving Policies, I confirm this Work has not been accepted for publication elsewhere, is not being considered for publication elsewhere and does not duplicate material already published. I confirm all authors consent to publication of this Work and authorise the granting of this licence.

For peer review only

Title

Development of an occupational advice intervention for patients undergoing elective hip and knee replacement: A Delphi study

Authors

- Paul Baker (1,2)
- Lucksy Kottam (1)
- Carol Coole (3)
- Avril Drummond (3)
- Catriona McDaid (2)
- Amar Rangan (1,2,4)

On behalf of the OPAL study group (see acknowledgments)

Institutions

1. Department of Trauma and Orthopaedics, South Tees Hospitals NHS Foundation Trust, Middlesbrough
2. York Trials Unit, Department of Health Sciences, University of York, York
3. School of Health Sciences, Faculty of Medicine & Health Sciences, University of Nottingham, Nottingham
4. Faculty of Medical Sciences & Nuffield Department of Orthopaedics Rheumatology and Musculoskeletal Sciences, University of Oxford, Oxford

Corresponding author information

Email: paul.baker1@nhs.net
Tel: 01642 854479

For peer review only

Abstract

Objective: To obtain consensus on the content and delivery of an occupational advice intervention for patients undergoing primary hip and knee replacement surgery. The primary targets for the intervention were 1) patients, carers and employers through the provision of individualised support and information about returning to work, and 2) hospital orthopaedic teams through the development of a framework and materials to enable this support and information to be delivered.

Design: Modified Delphi study as part of a wider intervention development study (The OPAL study: HTA Reference 15/28/02).

Setting: Five stakeholder groups (patients, employers, orthopaedic surgeons, General Practitioners (GPs), allied health professionals (AHPs) and nurses) recruited from across the United Kingdom.

Participants: Sixty-six participants.

Methods: Statements for the Delphi process were developed relating to the content, format, delivery, timing and measurement of an occupational advice intervention. The statements were based on evidence gathered through the OPAL study that was processed using an intervention mapping framework. Intervention content was examined in Round 1 and intervention format, delivery, timing and measurement were examined in Round 2. In Round 3 the developed intervention was presented to the stakeholder groups for comment.

Consensus: For Rounds 1 and 2 consensus was defined as 70% agreement or disagreement on a 4-point scale. Statements reaching consensus were ranked according to the distribution of responses to create a hierarchy of agreement. Round 3 comments were used to revise the final version of the developed occupational advice intervention.

Results: Consensus was reached for 36 of 64 Round 1 content statements (all agreement). In Round 2, 13 questions were carried forward and an additional 81 statements were presented. Of these, 49 reached consensus (44 agreement/5 disagreement). Eleven respondents provided an appraisal of the intervention in Round 3.

Conclusions: The Delphi process informed the development of an occupational advice intervention as part of a wider intervention development study. Stakeholder agreement was achieved for a large number of intervention elements encompassing the content, format, delivery and timing of the intervention. The effectiveness and cost-effectiveness of the developed intervention will require evaluation in a randomized controlled trial.

Keywords: Return to work, Hip replacement, Knee replacement, Occupational advice, Employment, Surgery

Strengths and limitations of this study:

- Wide stakeholder engagement including patients, employers, surgeons, GPs, Allied Health professionals with knowledge of the process of returning to work after hip or knee replacement
- Recruitment of a large number of Delphi members who were invited to participate in all 3 Delphi rounds

For peer review only

- Initial Delphi statements developed from research performed as part of the OPAL (Occupational advice for Patients undergoing Arthroplasty of the Lower limb) study (HTA 15/28/02).
- Loss of participants between each of the rounds of questioning, specifically the employer group who failed to participate in the final Delphi round.
- The process recruited participants from the United Kingdom only, which potentially limits the generalizability of findings to healthcare settings internationally.

For peer review only

Introduction

Hip and knee osteoarthritis are associated with a reduction in work participation and productivity and an increased risk of work loss. [1 2] The costs associated with occupational musculoskeletal disorders are significant .[3 4] The estimated annual cost of workplace ill health is £9.7 billion, equivalent to £18,400 per case.[5] These costs are borne not only by the individual (impact of ill health on quality of life), but also by their employers and society (loss of productivity, need for health care, rehabilitation and compensation).[3 4] In addition to the financial costs, lengthy sickness absence can result in work disability, poorer general health, increased risk of mental health problems and higher mortality.[6-8] Working therefore has physical and mental health benefits, alongside its socioeconomic value.

Lower limb joint replacement is an effective and cost-effective treatment for patients with hip and knee osteoarthritis.[9-12] Recent changes to the pension age combined with an ageing UK workforce have resulted in a steady increase in the numbers of hip and knee replacements being performed in patients of working age over the last decade .[13-15] In 2017, 18,812 (20.5% of all hip replacements) and 17,765 (17.4% of all knee replacements) were performed in patients aged less than 60 years.[13-15] Current recommendations supporting return to work after hip and knee replacement are limited and inconsistent.[16] There is variation in the content, delivery and format of occupational advice delivered to patients having hip and knee replacements and a need to provided more comprehensive, individualised advice for these patients to support early, sustained return to work after surgery.[16]

The OPAL study (Occupational advice for Patients undergoing Arthroplasty of the Lower limb) was a National Institute for Health Research – Health Technology Assessment (NIHR-HTA) commissioned research study that aimed to develop an occupational advice intervention to support return to work after hip and knee replacement.[16] OPAL used an intervention mapping framework supported by related qualitative and quantitative work streams.[16] Initial research evaluated the specific needs of the population of patients who were in work and intended to return to work following surgery, established how individual patients returned to work and documented the barriers preventing return to work.[17 18] Through these work streams a range of key performance indicators and potential intervention components that could be used to develop an occupational advice intervention emerged.

To refine these components and address areas of uncertainty relating to the intervention a multi-stakeholder intervention development group was constructed to ascertain whether agreement could be reached about the design, content, delivery, format and timing of the proposed occupational advice intervention. To facilitate this process a modified Delphi consensus process was employed.[16] The Delphi approach was chosen as it can be delivered remotely in a short timeframe without the need to convene meetings. It also enables researchers to collect the opinions of a range of different individuals with differing areas of expertise which was desirable in this setting. The initial research performed as part of the intervention mapping process provided the basis for this process by generating an initial list of statements for the Delphi consensus development.

For peer review only

Methods

Design of the modified Delphi study

A modified 3 round Delphi consensus process was used.[19-21] The process was guided by the information gathered from research completed during the first phase of the OPAL project.[16-18]

During the first phase of the OPAL project a number of intervention components emerged that were considered likely to be integral to the development of a successful occupational advice intervention (Table 1). Expanded versions of these components were used as the basis for initial statement development that could be explored during the Delphi process.

Delphi stakeholder recruitment

Five stakeholder groups were identified for inclusion in the modified Delphi process. The sampling strategy for each stakeholder group is outlined in Table 2, with participants chosen via a targeted approach to maximise patient, public and professional engagement. To ensure wide participation and the validity of the consensus process the process aimed to recruit a minimum of 5 individuals from each stakeholder group. A maximum limit of 15 individuals from any given stakeholder group was chosen to ensure one group's opinions did not overwhelm the opinions of others within the consensus process. As such, we aimed to have a minimum of 25 participants and a maximum of 75 participants for each round.

Although there are no definitive rules about the sample size for a Delphi study, a minimum of 8 to 10 participants has been suggested.[22] Whilst higher response rates and ease of administration are an advantage of smaller homogeneous groups, we considered a larger sample size desirable given the variation in expertise and the heterogeneity within our stakeholder groups. Furthermore, if areas of uncertainty are being explored larger sample sizes can help to reduce errors and improves the reliability of the findings.[23]

Prior to enrolment, potential participants from all stakeholder groups were invited to participate via an email from a member of the OPAL study team as per the sampling strategy for each stakeholder group outlined in Table 2. This email included a participant information sheet describing the Delphi consensus process and what participation involved. Participants were asked to confirm their consent to participate by return of email and only those that responded indicating their willingness to participate were included in the process.

Development of Delphi statements

Prior to commencing, statements relating to the proposed **content, format, delivery, timing and measurement** of an occupational advice intervention were developed. Due to the breadth of statements developed and their inter-related nature, we adopted a step-wise approach to the presentation of individual statements to the Delphi group. Round 1 focussed on defining the **content** of the intervention in 2 sections. Section 1, focussed on passive content ('written' advice and information) and section 2 on active content (actions or processes for patients, employers and healthcare members to undertake). These statements were piloted by a small sample of surgeons, GPs and patients. Having first defined the content, we then used this information to refine the statements relating to the format, delivery, timing and measurement of this content presented in Round 2. In Round 2 statements were grouped under headings allowing exploration around specific themes. Round 3 was then used to clarify any areas of residual uncertainty from Rounds 1 and 2 and

For peer review only

present the proposed occupational advice intervention back to the Delphi participants for final comments.

For each statement within the Delphi process participants were asked to rate the extent of agreement with individual statements about the importance of including specific elements in a occupational advice intervention, with possible options being: strongly agree; agree; disagree; strongly disagree; don't know. For a subset of statements in Round 1 they were also asked to rate the deliverability of the content or action alongside current healthcare provision. Therefore for some statements the participants were asked to provide two ratings one for 'importance' and one for 'deliverability'.

At the end of each section, there was a free text box where participants could add suggestions relating to the intervention that could be evaluated in subsequent rounds. In rounds where statements from a previous Delphi round were being re-presented, these were presented alongside controlled feedback with modal round one rating for these statements; the proportion of each response option selected by the other participants; and a reminder of the participant's own previous ratings.

Delivery of Delphi survey

The Delphi survey was delivered via email using an online web-based survey platform.[24] Round 1 was delivered between 25 September 2017 and 13 October 2017, Round 2 between 22 November 2017 and 13 December 2017 and Round 3 between 01 June 2018 and 22 June 2018. The email included a covering letter to the participants and an electronic link to the questionnaires. All 3 rounds allowed a minimum of 3 weeks for participants to respond. Automated reminders were sent via the electronic system after 10 days from the day of initialising the survey. A further personalised email reminder was sent to non-responders during the final week of the surveys.

Round 1 and 2 questionnaires required respondents to provide their initials and occupation. All Round 2 emails incorporated an overall report summarising the pooled responses from Round 1 survey and, where appropriate, the responses of each of the 5 stakeholder groups. In addition, for those participants who completed the Round 1 survey, an individualised report summarising their responses to the statements in Round 1 were included with the Round 2 survey to allow participants to reappraise their responses in view of the overall responses.[25] Round 3 emails included 4 core documents from the developed occupational advice intervention (a summary of the intervention, occupational checklist, patient 'return to work' workbook and employer booklet) for participants to review and comment. Email reminders were sent to non-responders during the final week of the surveys.

Analysis of data

Descriptive analyses of the Delphi responses were undertaken by the OPAL study team. Results of each round were discussed with the wider OPAL study research team before the statements were agreed for subsequent rounds.

An a priori consensus threshold of 70% (strongly agree/agree or strongly disagree/disagree) was agreed before statements were circulated.[25] There is no universal agreement on an acceptable level of consensus for a Delphi study,[26 27] however reports suggest this should be decided before commencing the study and recommends at threshold of at least 70% to ensure validity of the findings.[27] For statements that failed to reach consensus, further

For peer review only

analysis was undertaken based on responses for each of the 5 stakeholder subgroups. The following rules were then employed to determine which statements were discarded and which were re-presented in the next round.

- If no or only one stakeholder group reached concordant consensus ($\geq$70% agreement or disagreement) then the statement would be withdrawn
- If two or more stakeholder groups reached concordant consensus ($\geq$70% agreement or disagreement) then the statement would be re-presented in a subsequent round.
- In the situation where one or more stakeholder groups reach 'agreement' and another group reach 'disagreement' the statement would be discussed by the OPAL investigator team and a decision on inclusion / exclusion of the statement would be made.

For statements that were rated for both importance and deliverability in Round 1, consensus was reached if the 70% threshold was achieved for both the importance and deliverability rating. Statements that reached consensus for one of the domains were analysed by stakeholder group as described above.

In Rounds 1 and 2 statements reaching consensus were ranked according to the distribution of responses to create a hierarchy of agreement.

In Round 3 the occupational advice intervention and associated documents were circulated for comment. Descriptive open feedback from participants to these documents were recorded.

Ethics Approval-

This study was reviewed and given favourable opinion by the East Midlands - Derby Research Ethics Committee (16/EM/0341) and approved by the Health Research Authority in UK.

Patient and Public Involvement

The OPAL research project was developed in collaboration with members of the British Orthopaedic Association (BOA) Patient Liaison Group (PLG). A patient co-applicant from the BOA PLG was involved in the development of the research question and defining the outcome measures used within the wider OPAL study. Patients were involved in the design of the study from inception of the project, through protocol development, study delivery and project dissemination. These included patients from the BOA PLG, the National Joint Registry patient group and patient and public groups affiliated with the sponsor site.

For peer review only

Results

Round 1:

Responses were received from 43 of the 66 participants (65%) including 14 patients, 8 surgeons, 6 GPs, 11 allied health professionals and nurses, and 4 employers. In section 1 ('written' advice and information), consensus was reached for 26 of 32 statements (81%). Of the remaining 6 statements, 5 reached consensus for 2 or more stakeholder groups and were therefore taken forward to Round 2 and one statement was discarded. Section 1 statements reaching consensus and ranked based on the strength of consensus are listed in Table 3.

In section 2 (actions or processes for patients, employers and healthcare members to undertake), participants were asked to rate both the importance and deliverability of each statement. Of the 32 components presented only 10 (31%) reached consensus for both importance and deliverability (Table 4). Of the remaining 22 statements, 14 reached consensus for importance but not deliverability, 2 reached consensus for deliverability but not importance and 6 did not reach consensus for either. Of these statements 7 reached consensus for both importance and deliverability for 2 or more stakeholder groups and were therefore taken forward to Round 2 and 15 statements were discarded.

Round 2:

Responses were received from 26 of the 66 participants (39%) including 8 patients, 7 surgeons, 3 GPs, 6 allied health professionals and nurses, and 2 employers.

Twelve questions carried forward from Round 1 plus one additional question generated from the free text comments were presented to the participants. Of these, 10 reached consensus based on their potential importance within the proposed occupational advice intervention.

A further 81 statements grouped into 13 categories were then rated. This allowed the team to explore different approaches to a given problem. For example the first category asked participants to rate a set of 5 statements relating to which healthcare team member should have responsibility for delivery and co-ordination of the occupational advice intervention. If at least one or more statements in a given category reached consensus this was taken as representative of the Delphi group's position relating to the given category and the remaining statements were discarded. Overall 49 statements (60%) reached consensus (44 agreement and 5 disagreement), at least one statement in every category reached consensus (Appendix Table 1).

The Occupational advice intervention:

Based on the evidence gathered throughout the OPAL study and consolidated through Delphi rounds 1 and 2 the occupational advice intervention was further developed and finalised.

The intervention was designed to support patients throughout their surgical pathway, starting during their initial outpatient appointment and continuing until 16 weeks after surgery. It had a number of key themes that linked to performance objectives for patients and staff and was supported by a range of patient and staff resources (to support delivery

For peer review only

and measurement of the intervention). A simplified schematic of the OPAL 'return to work' intervention is presented in figure 1.

Round 3:

In Round 3, the finalised occupational advice intervention along with selected patient and staff materials were circulated to 65 of the 66 Delphi participants for comment (one patient withdrew). Responses were received from 11 participants comprising a constructive appraisal of the intervention from 9, as well as highlighting typographical and formatting issues. The feedback was positive in all cases.

A diagram of the overall Delphi consensus process is shown in figure 2.

For peer review only

Discussion

The Delphi consensus methodology was used to underpin the development of an evidence-based, theory-driven occupational advice intervention to assist patients returning to work after elective hip and knee replacement. It enabled the OPAL study team to rationalise the content, format, delivery and timing of the intervention and clarified areas of uncertainty related to the intervention that had arisen during the earlier stages of the research. Response to the developed intervention during the third round of the Delphi process was positive, validating the use of the Delphi process to support intervention development.

Prior to the Delphi process, the OPAL study had already completed a number of complementary research phases to enable the OPAL team understand the current evidence, stakeholder and patient perspectives, and current practice relating to return to work after hip and knee replacement.[16-18] Through the intervention mapping framework this is information generated a range of components for our intervention. The Delphi methodology was then used to 'refine' the intervention and reach consensus on the final design. This is similar to the modified Delphi approach used by Vonk Noordegraaf el al to develop a return to work intervention for gynaecological surgery, as it used existing evidence as the basis for the process but sought to bridge gaps and clarify uncertainty within this evidence.[28] However, one limitation of this approach is that it may inadvertently narrow the focus of the intervention with only components deemed important by the research team included. There is a risk that potentially useful intervention components that may have been of interest to the stakeholder groups were not included as the starting position was pre-defined. However, the approach used is not unusual and is similar to the approaches used by others.[20 29] Furthermore, given the breadth of work completed earlier in the OPAL study and the design of the modified Delphi survey allowing participants to suggest new intervention components within each round, this is unlikely to have had a negative impact.

Broad stakeholder involvement helped the research team ensure the final intervention was acceptable to all groups, increasing the chances of success when implemented and delivered. Unfortunately, despite good initial engagement, the response rates reduced as the process progressed. This is a common finding during Delphi processes[20] and was perhaps related to the larger sample size involved and extended period of the process with a 6 week gap between Round 1 and 2 and a 6 month gap between Rounds 2 and 3. The gap between Round 2 and 3 was necessary as the intervention needed to be finalised with associated materials being developed during this period. Other contributing factors may include the increasing length of the Delphi questionnaires with each round and the volume of materials that needed to be reviewed in Round 3. All participants were UK based and working with the setting of the UK National Health Service and social care provision or UK employment. Therefore, this may impact on the generalisability of the findings outside of the UK health setting.

While the low response rate in Round 3 may be a concern, the purpose of this round was to circulate and draw comment regarding the final intervention rather than reach consensus on specific points. With 11 respondents, including at least one member from each stakeholder group, this seems valid as Delphi process relies more on the group dynamics even with reaching consensus rather than their statistical power and a lower limit of 10 participants is often considered sufficient for a Delphi panel.[30 31] During the process there was a notable drop off in employer respondents. In total 12 employers initially expressed an interest in participating, however only four responded in round 1, two in round 2 and one in round 3. It is often difficult to engage employers in research[32] and despite using a number of

For peer review only

complementary strategies (17) we were unable to maintain engagement. However, as the intervention was designed to be delivered in secondary care rather than in the workplace this potentially did not significantly influence the nature of the final intervention.

The modified Delphi methodology employed in this study resolved uncertainties about a number of intervention components. However, there were a few areas where the consensus process was limited. Two key areas that stakeholders felt were important were 1) the provision of additional pre and post-operative physiotherapy/occupational therapy (over and above standard care) in which return to work issues could be addressed and 2) the identification of 'high risk' patients that should be provided with additional help and support. Yet, these positions conflicted with other information gathered from the Delphi participants and the evidence from OPAL phase 1. Essentially firstly, our cohort study failed to identify a 'high risk' population and the current literature describing predictors of return to work after hip and knee replacement was limited.[33-38] This meant we were not able to confidently identify a 'high risk' group in need of a more intensive targeted intervention. Secondly, there was concern about the cost, time and logistics associated with the implementation of a resource intensive intervention requiring additional patient interactions. The survey of practice and stakeholder / patient interviews demonstrated that services varied significantly in their structure and the resources available.[18] To be successful it was agreed that the intervention should supplement rather than replace existing pathways of care and should, where possible, use existing staff and adapt current working. Comments from Delphi participants, the OPAL research team and the study steering committee had similarly raised concerns about the implementation and sustainability of an intervention requiring significant additional resources. The OPAL research team therefore felt that, despite this component reaching consensus, it was prudent to pursue a less intensive model to improve implementation of the final intervention.

We were unable to compare our intervention to other occupational advice interventions for patients undergoing hip and knee replacement as no such interventions have been reported in the literature. A rapid evidence synthesis performed earlier in the OPAL study (PROSPERO protocol registration number CRD42016045235) found only four studies that reported occupational advice interventions for patients undergoing elective surgery. This included two randomised clinical trials from Belgium and the Netherlands in patients undergoing gynaecological surgery and lumbar disc surgery [39 40] and two qualitative studies that explored factors affecting return to work from the perspective of the patient following knee replacement[41] and factors influencing work disability following mastectomy[42]. Of the two interventions described in the RCTs one described a personalised eHealth intervention[39] whereas the other assessed a rehabilitation-orientated intervention focussing on early resumption of activities.[40] Our intervention drew on elements of both of these interventions in terms of delivering an individualised patient-centred approach while encouraging early resumption of workplace activities through discussion with employers alongside workplace adaptions and alterations to working patterns.

In conclusion, a modified Delphi consensus process employed within a wider intervention development project facilitated the development of the OPAL occupational advice intervention. Consensus was reached for a range of intervention components that allowed the content, format, delivery and timing of the intervention to be finalised. The intervention developed and the materials created to support its delivery were well received by the Delphi group. The effectiveness and cost-effectiveness of the developed intervention will require evaluation in a randomised controlled trial.

For peer review only

OPAL Collaborators

The following researchers contributed to the concept, design, delivery and analysis of the OPAL study:

Sayeed Khan (Make UK, the Manufacturers Organisation), Louise Thomson (Faculty of Medicine and Health Sciences, University of Nottingham), Catherine Hewitt and Catriona McDaid (York Trials Unit, University of York), Iain McNamara (Norfolk and Norwich University Hospital NHS Foundation Trust), David McDonald (University of Stirling), Judith Fitch (British Orthopaedic Association Patient Liaison Group), Gerry Richardson (Centre for Health Economics, University of York)

The authors acknowledge the contributions of other members of the wider OPAL study team: Reece Walker and Amanda Goodman (South Tees Hospitals NHS Foundation Trust); Sarah Ronaldson and Elizabeth Coleman (York University); Dr Fiona Nouri and Dr Melanie Narayanasamy (University of Nottingham).

The OPAL investigators acknowledge the support of the British Orthopaedic Association Orthopaedic Surgery Research Centre (BOSRC) which supports the development of surgical trials.

Funding acknowledgement

This project was funded by the National Institute for Health Research Health Technology Assessment (HTA) programme (project number 15/28/02). Further information available at https://www.journalslibrary.nihr.ac.uk/programmes/hta/152802/#/

The funding body have not been involved in the design of the study, data collection, data analysis and data interpretation. They have not been involved in the writing of this manuscript.

Department of Health and Social Care disclaimer

This report presents independent research commissioned by the National Institute for Health Research (NIHR). The views and opinions expressed by the authors in this publication are those of the authors and do not necessarily reflect those of the National Health Service, the National Institute for Health Research, Medical Research Council, Central Commissioning Facility, NIHR Evaluations, Trials and Studies Coordinating Centre, the Health Technology Assessment Programme or the Department of Health.

Competing interests

Amar Rangan is supported by the National Institute for Health Research (NIHR) Oxford Biomedical Research Centre (BRC). Catriona McDaid is a member of the NIHR HTA and EME Editorial Boards. The other authors declare they have no competing interests

Author Contributions

- Paul Baker: If the Chief Investigator for the OPAL study. Contributed to the conception of the study, the design of the work; the acquisition, analysis, or interpretation of data for the work; drafted the manuscript.
- Lucksy Kottam: Contributed to the design of the work; the acquisition, analysis, or interpretation of data for the work; drafted the manuscript.
- Carol Coole: Contributed to the conception of the study, the design of the work; the interpretation of data for the work; edited the manuscript.
- Avril Drummond: Contributed to the conception of the study, the design of the work; the interpretation of data for the work; edited the manuscript.

For peer review only

- Catriona McDaid: Contributed to the conception of the study, the design of the work; the interpretation of data for the work; edited the manuscript.
- Amar Rangan: Contributed to the design of the work; the acquisition, analysis, or interpretation of data for the work; drafted the manuscript.

Data Availability

Anonymised data detailing the individual participants responses within each round of the OPAL Delphi study is available upon request. Please submit requests in writing to the Department of Research and Innovation, South Tees Hospitals NHS Foundation Trust, Marton Rd, Middlesbrough, UK.

For peer review only

References

1. Bieleman HJ, Bierma-Zeinstra SM, Oosterveld FG, et al. The effect of osteoarthritis of the hip or knee on work participation. *The Journal of rheumatology* 2011;38(9):1835-43. doi: 10.3899/jrheum.101210 [published Online First: 2011/06/17]
2. Sharif B, Garner R, Sanmartin C, et al. Risk of work loss due to illness or disability in patients with osteoarthritis: a population-based cohort study. *Rheumatology (Oxford, England)* 2016;55(5):861-8. doi: 10.1093/rheumatology/kev428 [published Online First: 2016/01/14]
3. Bhattacharya A. Costs of occupational musculoskeletal disorders (MSDs) in the United States. *International Journal of Industrial Ergonomics* 2014;44(3):448-54. doi: https://doi.org/10.1016/j.ergon.2014.01.008
4. Executive THaS. Lower limbs: Information for employees 2018 [Available from: http://www.hse.gov.uk/msd/lld/employees.htm accessed 14/04/2020.
5. Executive THaS. Costs to Great Britain of workplace injuries and new cases of work-related Ill Health – 2017/18 [Available from: https://www.hse.gov.uk/statistics/cost.htm accessed 14/04/2020.
6. Gignac MA, Badley EM, Lacaille D, et al. Managing arthritis and employment: making arthritis-related work changes as a means of adaptation. *Arthritis and rheumatism* 2004;51(6):909-16. doi: 10.1002/art.20822 [published Online First: 2004/12/14]
7. O'Brien GE, Feather NT. The relative effects of unemployment and quality of employment on the affect, work values and personal control of adolescents. *Journal of Occupational Psychology* 1990;63(2):151-65. doi: 10.1111/j.2044-8325.1990.tb00517.x
8. Waddell G BA. Is work good for your health and well-being? . London (UK): The Stationery Office, 2006.
9. Jones CA, Voaklander DC, Johnston DW, et al. Health related quality of life outcomes after total hip and knee arthroplasties in a community based population. *The Journal of rheumatology* 2000;27(7):1745-52. [published Online First: 2000/07/29]
10. Ethgen O, Bruyere O, Richy F, et al. Health-related quality of life in total hip and total knee arthroplasty. A qualitative and systematic review of the literature. *The Journal of bone and joint surgery American volume* 2004;86-a(5):963-74. [published Online First: 2004/05/01]
11. Fitzgerald JD, Orav EJ, Lee TH, et al. Patient quality of life during the 12 months following joint replacement surgery. *Arthritis and rheumatism* 2004;51(1):100-9. doi: 10.1002/art.20090 [published Online First: 2004/02/12]
12. Bachmeier CJ, March LM, Cross MJ, et al. A comparison of outcomes in osteoarthritis patients undergoing total hip and knee replacement surgery. *Osteoarthritis and cartilage* 2001;9(2):137-46. [published Online First: 2001/05/02]
13. Registry NJ. Hips - Primary Procedures - Patient Characteristics- Age and gender of primary hip replacement patients. 2018. https://reports.njrcentre.org.uk/hips-primary-procedures-patient-characteristics.
14. Registry NJ. Knees - Primary Procedures - Patient Characteristics- Age and gender of primary knee replacement patients 2018. https://reports.njrcentre.org.uk/knees-primary-procedures-patient-characteristics.
15. Registry NJ. NJR 15th Annual Report; National Joint Registry for England, Wales, Northern Ireland and the Isle of Man, Surgical data to 31 December 2017 2018. https://reports.njrcentre.org.uk/Portals/7/PDFdownloads/NJR%2015th%20Annual%20Report%202018.pdf.

For peer review only

16. Baker P, Coole C, Drummond A, et al. Development of an occupational advice intervention for patients undergoing lower limb arthroplasty (the OPAL study). *BMC Health Services Research* 2018;18(1):504. doi: 10.1186/s12913-018-3238-z
17. Coole C, Nouri F, Narayanasamy M, et al. Engaging workplace representatives in research: what recruitment strategies work best? *Occupational medicine (Oxford, England)* 2018;68(4):282-85. doi: 10.1093/occmed/kqy047 [published Online First: 2018/03/27]
18. Nouri F, Coole C, Narayanasamy M, et al. Managing Employees Undergoing Total Hip and Knee Replacement: Experiences of Workplace Representatives. *Journal of Occupational Rehabilitation* 2019;29(2):451-61. doi: 10.1007/s10926-018-9805-7
19. Jones J, Hunter D. Consensus methods for medical and health services research. *Bmj* 1995;311(7001):376-80. [published Online First: 1995/08/05]
20. Sinha IP, Smyth RL, Williamson PR. Using the Delphi technique to determine which outcomes to measure in clinical trials: recommendations for the future based on a systematic review of existing studies. *PLoS medicine* 2011;8(1):e1000393. doi: 10.1371/journal.pmed.1000393 [published Online First: 2011/02/02]
21. Murphy MK, Black NA, Lamping DL, et al. Consensus development methods, and their use in clinical guideline development. *Health Technol Assess* 1998;2(3):i-iv, 1-88. [published Online First: 1998/04/30]
22. Hallowell MR, Gambatese JA. Qualitative Research: Application of the Delphi Method to CEM Research. *Journal of Construction Engineering and Management* 2010;136(1):99-107. doi: doi:10.1061/(ASCE)CO.1943-7862.0000137
23. Wilkes L. Using the Delphi technique in nursing research. *Nursing standard (Royal College of Nursing (Great Britain) : 1987)* 2015;29(39):43-9. doi: 10.7748/ns.29.39.43.e8804 [published Online First: 2015/05/28]
24. SurveyMonkey Inc San Mateo, California, USA [Available from: www.surveymonkey.com accessed 14/04/2020.
25. Sinead Keeney FH, Hugh McKenna. The Delphi Technique in Nursing and Health Research: John Wiley & Sons Ltd, The Atrium, Southern Gate, Chichester, West Sussex, PO19 8SQ, United Kingdom 2011.
26. Hasson F, Keeney S, McKenna H. Research guidelines for the Delphi survey technique. *Journal of advanced nursing* 2000;32(4):1008-15. [published Online First: 2000/11/30]
27. Keeney S, Hasson F, McKenna H. Consulting the oracle: ten lessons from using the Delphi technique in nursing research. *Journal of advanced nursing* 2006;53(2):205-12. doi: 10.1111/j.1365-2648.2006.03716.x [published Online First: 2006/01/21]
28. Vonk Noordegraaf A, Huirne J, Brölmann H, et al. Multidisciplinary convalescence recommendations after gynaecological surgery: a modified Delphi method among experts. *BJOG: An International Journal of Obstetrics & Gynaecology* 2011;118(13):1557-67. doi: 10.1111/j.1471-0528.2011.03091.x
29. Slade SC, Dionne CE, Underwood M, et al. Standardised method for reporting exercise programmes: protocol for a modified Delphi study. *BMJ Open* 2014;4(12):e006682. doi: 10.1136/bmjopen-2014-006682
30. Okoli C, Pawlowski SD. The Delphi method as a research tool: an example, design considerations and applications. *Information & Management* 2004;42(1):15-29. doi: https://doi.org/10.1016/j.im.2003.11.002
31. Akins RB, Tolson H, Cole BR. Stability of response characteristics of a Delphi panel: application of bootstrap data expansion. *BMC medical research methodology* 2005;5(1):37. doi: 10.1186/1471-2288-5-37

For peer review only

32. Lysaght R, Kranenburg R, Armstrong C, et al. Participant Recruitment for Studies on Disability and Work: Challenges and Solutions. *J Occup Rehabil* 2016;26(2):125-40. doi: 10.1007/s10926-015-9594-1 [published Online First: 2015/07/17]
33. Tilbury C, Schaasberg W, Plevier JW, et al. Return to work after total hip and knee arthroplasty: a systematic review. *Rheumatology (Oxford, England)* 2014;53(3):512-25. doi: 10.1093/rheumatology/ket389 [published Online First: 2013/11/26]
34. Kuijer PPF, de Beer MJP, Houdijk JHP, et al. Beneficial and limiting factors affecting return to work after total knee and hip arthroplasty: a systematic review. *Journal of Occupational Rehabilitation* 2009;19(4):375-81. doi: 10.1007/s10926-009-9192-1
35. Sankar A, Davis AM, Palaganas MP, et al. Return to work and workplace activity limitations following total hip or knee replacement. *Osteoarthritis and cartilage* 2013;21(10):1485-93. doi: 10.1016/j.joca.2013.06.005 [published Online First: 2013/06/19]
36. Tilbury C, Leichtenberg CS, Tordoir RL, et al. Return to work after total hip and knee arthroplasty: results from a clinical study. *Rheumatology international* 2015;35(12):2059-67. doi: 10.1007/s00296-015-3311-4 [published Online First: 2015/06/30]
37. Leichtenberg CS, Tilbury C, Kuijer P, et al. Determinants of return to work 12 months after total hip and knee arthroplasty. *Annals of the Royal College of Surgeons of England* 2016;98(6):387-95. doi: 10.1308/rcsann.2016.0158 [published Online First: 2016/05/04]
38. Scott CEH, Turnbull GS, MacDonald D, et al. Activity levels and return to work following total knee arthroplasty in patients under 65 years of age. *The bone & joint journal* 2017;99-b(8):1037-46. doi: 10.1302/0301-620x.99b8.Bjj-2016-1364.R1 [published Online First: 2017/08/05]
39. Vonk Noordegraaf A, Anema JR, van Mechelen W, et al. A personalised eHealth programme reduces the duration until return to work after gynaecological surgery: results of a multicentre randomised trial. *BJOG : an international journal of obstetrics and gynaecology* 2014;121(9):1127-35; discussion 36. doi: 10.1111/1471-0528.12661 [published Online First: 2014/02/12]
40. Donceel P, M. BD, \par DL. Return to work after surgery for lumbar disc herniation - A rehabilitation-oriented approach in insurance medicine\par. *Spine 1999; 24(9 (May 1)): 872-876 \par* 1999
41. Bardgett M, Lally J, Malviya A, et al. Return to work after knee replacement: a qualitative study of patient experiences. *BMJ Open* 2016;6(2):e007912. doi: 10.1136/bmjopen-2015-007912
42. Hinman MR. Factors influencing work disability for women who have undergone mastectomy. *Women & Health* 2001;34(2):45-60.

For peer review only

Tables

Key intervention components identified during OPAL phase 1
• Education and advice<br>• Vocational counselling and guidance<br>• Physical therapy and exercises<br>• Work simulation/work hardening and job accommodation<br>• Contact with employer/workplace visits<br>• Multidisciplinary team involvement

Table 1: Key intervention components identified during OPAL phase 1

For peer review only

Stakeholder group	Requirement for inclusion	Participants recruited via:
Patients	Experience of returning to work after hip or knee replacement in the previous 12 months	• National Joint Registry patient network<br>• British Orthopaedic Association patient group<br>• Patient participants from OPAL phase 1
Employers and occupation health services	Experience of managing an employee returning to work after hip or knee replacement in the previous 12 months	• Federation of Small Businesses<br>• EEF – The manufacturers organisation<br>• Confederation of British Industry<br>• Trade Union Congress<br>• Department for Work and Pensions<br>• The Fit for Work Service<br>• The Work Foundation<br>• The Society of Occupational Medicine<br>• Institution of Occupational Safety & Health<br>• Society of Occupational Health Nurses
Orthopaedic Surgeons	Surgeons undertaking a minimum of 20 hip or knee replacements per year	• British Hip Society<br>• British Association for Surgeon of the Knee<br>• British Orthopaedic Association<br>• National Joint Registry
Allied Health Professionals (AHPs – Physiotherapists and Occupational therapists) and nurses	AHPs actively involved in the assessment and / or management of patients undergoing hip or knee replacement	• Association of Chartered *Physiotherapists* in *Occupational* Health and Ergonomics<br>• Chartered Society of Physiotherapy<br>• Occupational therapy networks e.g. College of Occupational Therapists Specialist Sections in Work and Trauma & Orthopaedics<br>• Royal College of nursing
General Practitioners	Experience of managing a patient returning to work after hip or knee replacement in the previous 12 months	• Local Medical Committees<br>• Royal College of General Practitioners<br>• Local Clinical Commissioning Groups

Table 2: Sampling strategy used to identify Delphi members

For peer review only

Is it important that an occupational advice intervention commenced prior to hip or knee replacement includes the following:	Agreement (%)
Q9. Information about exercises and rehabilitation following surgery	100%
Q13. Information about returning to driving	100%
Q3. A broad overview written for all stakeholders, of what to expect following surgery (rates and timing of expected recovery)	98%
Q15. Information about managing pain, types of analgesia and side effects	98%
Q5. Information about post-operative precautions, restrictions and activities to avoid following surgery	95%
Q18. Information about symptom management in relation to return to work and specific occupations e.g. expected levels of fatigue, pain, swelling	95%
Q12. Tips and tricks to help the patient manage around their home with day to day activities immediately following surgery	95%
Q10. Information regarding post-operative complications and their management	95%
Q14. Signposting to DVLA guidance	95%
Q23. Information for the patient about who to ask if they are having a problem returning to work	93%
Q4. Information about expected level of function at different time - points following surgery	88%
Q29. Advices about adaptions to working patterns to assist return including the use of phased returns, modified hours and altered work schedules	88%
Q21. Information and resources to support self-advocacy and empowerment	88%
Q20. Information about when it might be appropriate for patients and employers to access occupational health services	88%
Q19. Information for patients and employers about how to access occupational health services	88%
Q28. A list of potential workplace modifications, aids and adjustments that could be used to assist with return to work, with examples	84%
Q27. Information for the patients about how to ask for help at work from their employer and colleagues	84%
Q31. Guidance on how to set an appropriate provisional return to work date based on the date and type of surgery	81%
Q16. Guidance for orthopaedic care teams and G.Ps on how to use and prescribe a fit note	81%
Q24. Signposts to national and local support services e.g. Fit4Work, Citizens advices, ACAS	81%
Q11. Information about how having surgery may impact on social relationships	81%
Q32. Advice about how psychosocial and emotional factor influence return to work	79%
Q6. Information about how long the hip and knee replacement prostheses will last	77%
Q17. Examples of the correct use of fit notes	77%
Q30. A list of potential return to work barriers for patients and employers to consider	74%
Q8. Information about managing more than one joint replacement in close succession	72%

Table 3: Statements descriptions reaching consensus for section 1 (Ordered by % of respondents that strongly agreed or agreed)

For peer review only

How important / deliverable do you believe the following components are if an occupational advice intervention commencing prior to hip or knee replacement were to be developed	Agreement (%)	Agreement (%)
**10 statements reaching consensus for both IMPORTANCE and DELIVERABILITY**		
Q37. A post-operative mechanism for the identification of patients that are not progressing toward return to work as planned	95%	71%
Q52. Guidance for health services defining 'best practice' for patients returning to work after hip and knee replacement surgery	93%	82%
Q45. Training for members of the hospital orthopaedic care team to increase awareness about return to work issues	88%	82%
Q42. Interaction between the healthcare team and patient by phone, email or 'on-line' so that members of the care team can monitor progress and help the patient use the advice and information provided	88%	70%
Q64. Guidance on when in the return to work process patients can safely be discharged back to primary care for continued management of their return to work	86%	80%
Q36. A mechanism for pre-operative identification of patients at 'high risk' of prolonged sickness absence following surgery	86%	74%
Q51. Routine pre-operative therapy assessment during which a return to work plan is developed between the patients and the hospital orthopaedic care team	84%	80%
Q40. A separate intervention for hip and knee replacement patients that are not progressing towards return to work as planned	84%	79%
Q62. A process by which work status can be included in referral information for all patients referred from primary care into secondary care for consideration of hip or knee replacement	79%	79%
Q57. Information from patients that have experienced the process of returning to work after hip or knee replacement within the pre-operative education process	76%	73%

Table 4: Statements descriptions reaching consensus for section 2 (Ordered by % of respondents that strongly agreed or agreed)

For peer review only

Figure Legends

Figure 1: Simplified schematic of the OPAL 'return to work' intervention

Figure 2: Overview of the OPAL Delphi process

For peer review only

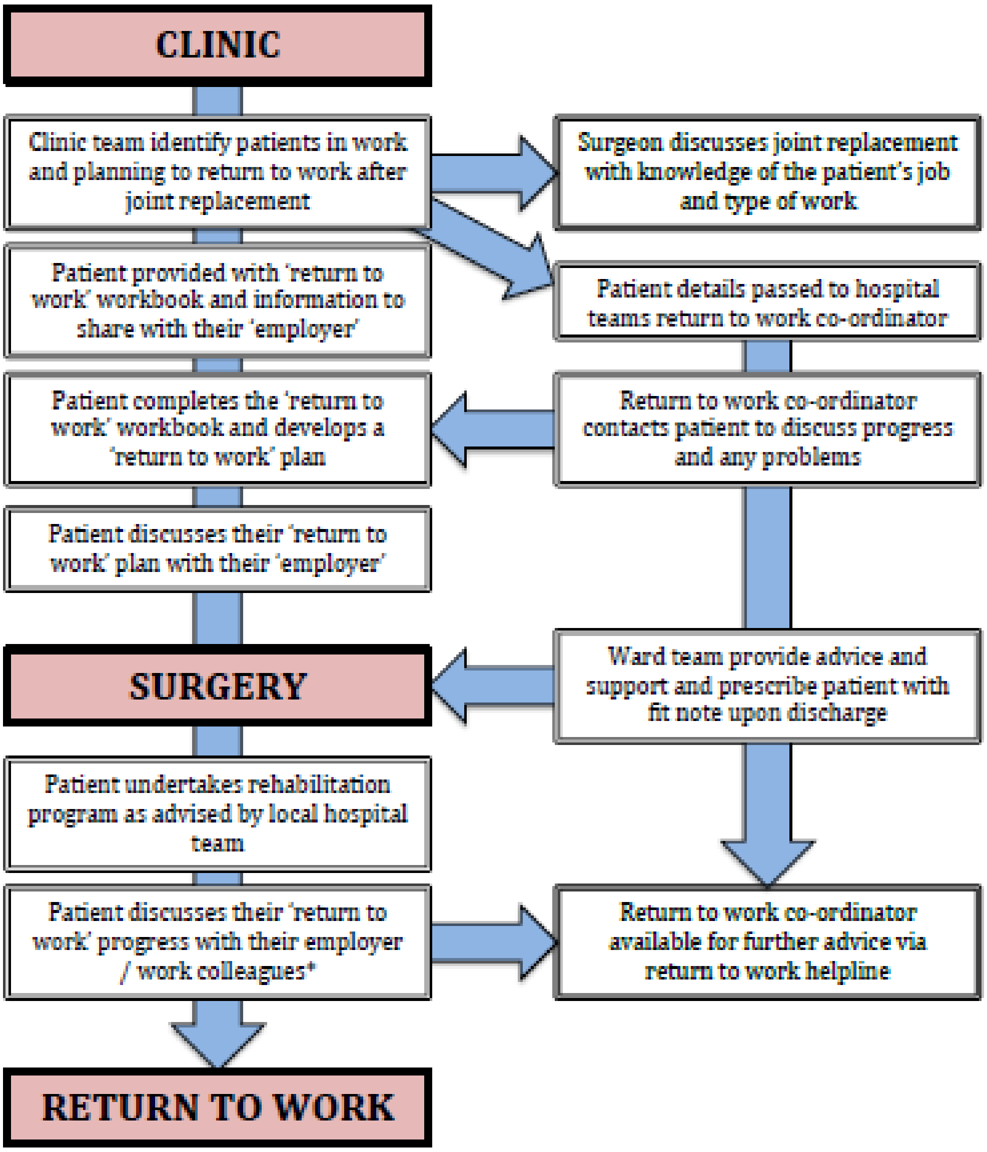

Figure 1: Simplified schematic of the OPAL 'return to work' intervention

Figure 2: Overview of the OPAL Delphi process

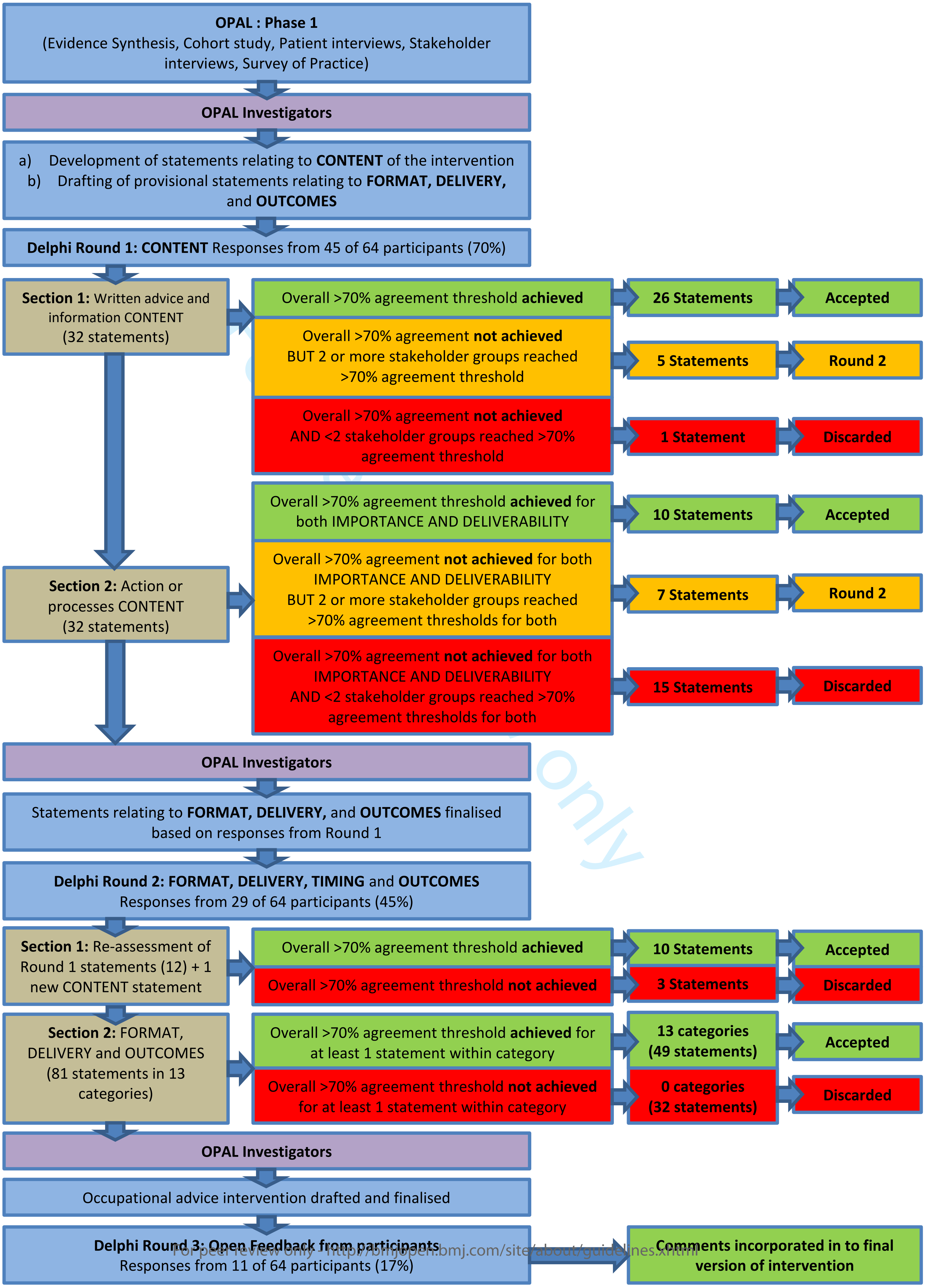

For peer review only - http://bmjopen.bmj.com/site/about/guidelines.xhtml

Appendices

	Category	Statements reaching consensus
1	Responsibility for delivery and co-ordination of the return to work intervention	Individual orthopaedic surgeons should be responsible for co-ordinating the return to work intervention for their own patients (88% disagreement)
2	Pre-operative identification of patients at 'higher risk' of prolonged sickness absence following surgery that may require additional individualised help and support	A face-to-face assessment with a staff member trained in the return to work intervention should be used to identify patients at 'higher risk' of prolonged sickness absence following surgery (80% agreement)
3	Pre-operative needs assessment	Hospital physiotherapy teams should see all patients intending to return to work prior to their surgery (80% agreement)<br><br>Hospital occupational therapy teams should see all 'higher risk' patients intending to return to work prior to their surgery (88% agreement)<br><br>Hospital physiotherapy team should see all 'higher risk' patients intending to return to work prior to their surgery (80% agreement)<br><br>Development of a return to work plan should be a routine part of the pre-assessment process for all patients undergoing hip and knee replacement (80% agreement)<br><br>Development of a return to work plan should be a routine part of the pre-assessment process for all 'higher risk' patients undergoing hip and knee replacements (96% agreement)<br><br>Pre-operative education sessions conducted by the hospital orthopaedic team should routinely include information about returning to work after surgery (88% agreement)<br><br>Patients should be encouraged to have a meeting with their employer prior to surgery to discuss their recovery and plans for returning to work (100% agreement)
4	Post-operative identification of patients at risk of an extended period off work after surgery	All return to work patients should be offered a routine physiotherapy review after discharge from hospital to help monitor their progress and identify those patients at risk of an extended period off work after surgery (84% agreement)<br><br>All return to work patients should be contacted by a member of the therapy team after discharge to help monitor their progress and identify those patients at risk of an extended period off work after surgery (92% agreement)<br><br>The hospital should provide a means of contact (phone, email etc) through which patients in need of additional help and support can contact the hospital orthopaedic team (92% agreement)<br><br>Patients should be encouraged to have a meeting with their employer in the 4 weeks following surgery to discuss their recovery and plan for return to work (72% agreement)
5	Additional care for Patients identified as 'higher risk' of an extended period off work after surgery	Additional post-operative physiotherapy review with a physiotherapist until specific work related rehabilitation targets have been achieved (92% agreement)<br><br>Additional post-operative occupational therapy review (76% agreement)<br><br>Ongoing therapy involvement until 'return to work' has been achieved

For peer review only

		(76% agreement)<br><br>Access to additional procedure specific information and advice (92% agreement)<br><br>Referral to the Fit4Work service (84% agreement)
6	Scope of training for staff	All members of the hospital orthopaedic team involved in the treatment of hip and knee replacement patients should receive training regarding the return to work intervention (76% agreement)<br><br>Specific staff members with responsibility for delivering 'return to work' advice should receive training regarding the return to work intervention (96% agreement)<br><br>All members of the therapy teams treating hip and knee replacement patients should receive training regarding the return to work intervention (88% agreement)<br><br>Training regarding the return to work intervention should be offered to local G.P groups (72% agreement)
7	Communicating occupational status and progress between stakeholders	The details of the patient's occupational status should be recorded in the primary care referral letter (96% agreement)<br><br>Details of the patient's occupational status should be confirmed and recorded in all interactions with secondary care (e.g. pre-operative outpatient appointments, discharge summaries post-operative follow-up appointment letters) (84% agreement)<br><br>Any information developed with the healthcare team as part of a patient return to work plan should be made available to the G.P (96% agreement)<br><br>Any information developed with the healthcare team as part of a patient return to work plan should be made available to the patient's employer and/or occupational health provider with their consent (84% agreement)<br><br>It is the patients and not the healthcare teams responsibility to communicate with their employer about their return to work (76% agreement)<br><br>All post-operative assessments by members of the therapy team should generate a statement about the patient's current functional status and estimated timeframe to return to work that is given to the patient and forwarded to their G.P (84% agreement)
8	Fit Notes	Fit notes should be completed in accordance with Department for Work and Pensions Fit Note Guidance (96% agreement)<br><br>Hospital orthopaedic departments should actively ask each and every patient if they require a fit note prior to discharge (96% agreement)<br><br>To discourage extended periods off work patients should be issued with a basic fit note excusing them from work for 2 weeks. Further fit notes can be issued based on progress towards recovery and access to phased return or amended duties and hours (72% disagreement)<br><br>G.Ps should be responsible for administering subsequent fit notes based on progress reports from the hospital orthopaedic team (76% agreement)
9	Format and delivery of patient information	All of the information and advice components relevant to patients should be included within a written patient booklet that is given to all patients. Information for other stakeholders will be delivered separately (76% agreement)<br><br>Only the most important (based on the ranking listed previously) information and advice components relevant to patients should be

For peer review only

		included within a written patient booklet that is given to all patients. Additional information could be made available via a website (80% agreement)<br><br>The patient booklet should include a section within which the patient and other stakeholders can record progress and feedback. This information would be held by the patient and could be shared with their permission (80% agreement)<br><br>The patient booklet should include a section within which the patient can record the outcome of any meetings and with their employer and/or occupational health team. This information would be held by the patient and could be shared with their permission (92% agreement)<br><br>Patients with previous experience of returning to work after joint replacement should be involved in the drafting of any patient facing materials (92% agreement)<br><br>The occupational advice intervention should include the ability for local hospital department to upload their own policies and procedures relating to post-operative recovery and return to work (92% agreement)<br><br>Primary Care Health professional and support staff should be able to direct potential surgical candidates to return to work information and advice prior to referral (88% agreement)
10	When should the intervention commence?	The intervention should commence in secondary care after the patient has had their operation but prior to discharge (88% disagreement)
11	Defining return to work	A patient has 'returned to work' only when they return to the same job (usual hours and duties) and activities outside of work they were doing prior to surgery (72% disagreement)
12	The aim of the intervention	The return to work interventions primary aim should be to return the patient to their pre-operative work role / level of occupational performance. (76% agreement)
13	Measuring return to work	The measurement of return to work should be graded based on specific work milestones for example return to place of work, return to normal hours, return to normal workplace activities (92% agreement)<br><br>The measurement of return to work should be based on resumption of specific work related activities (e.g. climbing, lifting, manual tasks) based on a list of pre-operative workplace activities (72% agreement)<br><br>The measurement of return to work is a binary measure – the patient has either returned to work or they have not (72% disagreement).<br><br>The rates of further sick leave in the 6 months after the patient has returned to work once is an important outcome measure (80% agreement)<br><br>The rates (type and duration) of any occupational adjustments implemented by the employer is an important outcome measure (84% agreement)<br><br>Asking patients about how they feel about their return to work is important outcome measure (96% agreement)<br><br>An assessment of whether or not the patient's preoperative occupational expectations following surgery have been met is an important outcome measure (92% agreement)

Appendix Table 1: Statements descriptions reaching consensus for Round 2

For peer review only
